# Machine Learning for Selecting High-Energy Phosphate Cathode Materials

**DOI:** 10.34133/research.0794

**Published:** 2025-07-29

**Authors:** Yongchun Dang, Zechen Li, Yongchao Yu, Xiwei Bai, Li Wang, Xuelei Wang, Peng Liu, Chen Sun, Xunli Zhou, Zhenpo Wang, Yongjie Zhao, Xiangming He, Lei Li

**Affiliations:** ^1^ National Engineering Research Center of Electric Vehicles, Beijing Co-innovation Centre for Electric Vehicles, Beijing Institute of Technology, Beijing 100081, China.; ^2^ Beijing Key Laboratory of Construction Tailorable Advanced Functional Materials and Green Applications, School of Materials Science and Engineering, Beijing Institute of Technology, Beijing 100081, China.; ^3^ Institute of Automation, Chinese Academy of Sciences, Beijing 100190, China.; ^4^ Institute of Nuclear and New Energy Technology, Tsinghua University, Beijing 100084, China.; ^5^ Beijing Institute of Technology Chongqing Innovation Center, Chongqing 401120, China.

## Abstract

The limited energy density inherent in cathode materials remains a marked barrier to the widespread adoption of sodium-ion batteries. Despite considerable research efforts, the precise influence of atomic and crystalline configurations on energy density is not yet fully understood, creating a knowledge gap that hinders the rational design of advanced cathode materials. In this study, we propose a machine learning approach to systematically identify promising cathode materials with high energy densities. Our model highlights the critical roles of entropy and equivalent electronegativity, among other properties such as molecular mass, electron affinity, and average ionic radius. Based on these insights, we successfully synthesized Na_3_Mn_0.5_V_0.5_Ti_0.5_Zr_0.5_(PO_4_)_3_ (NMVTZP) electrodes via a sol–gel method. The resulting electrodes exhibit an impressive reversible specific capacity of 148.27 mAh g^−1^ at a 0.1-C rate, outperforming several previously reported cathode materials. Additionally, the NMVTZP electrodes demonstrate an average operating voltage of 3.14 V, an energy density of 465 Wh kg^−1^, and exceptional rate performance, retaining 90.20 mAh g^−1^ at a 5-C rate. We anticipate that our machine learning approach will accelerate the development of high-performance materials and greatly contribute to the advancement of sodium-ion battery technology.

## Introduction

Rechargeable batteries are indispensable for the operation of electric vehicles (EVs) and grid storage systems [1–3]. Owing to their abundant raw materials, cost-effective production, and outstanding low-temperature functionality [4], sodium-ion batteries (SIBs) stand out as a promising option within the spectrum of electrochemical energy storage technologies [5,6]. Nevertheless, the limited energy density presents a marked hurdle to the broader application of SIBs. One of the most efficient strategies to address this issue is the optimization of cathode materials [7–12]. Polyanionic compounds, characterized by their expansive 3-dimensional frameworks, facilitate rapid Na^+^ insertion and extraction, thereby demonstrating excellent cycling and rate capabilities. Among these, sodium ion superionic conductors (NASICON) have garnered substantial attention in recent years due to their superior electrochemical attributes and structural robustness [13–15]. For example, Na_3_V_2_(PO_4_)_3_ boasts a reversible theoretical capacity of 117 mAh g^−1^ and exceptional cycling stability with over 3,000 cycles at high current rates [16]. Despite these advantages, the limited utilization of multi-electron reactions and the excessive reliance on toxic vanadium compounds impede its commercial viability. Consequently, extensive research has been directed toward unlocking the full potential of NASICON cathodes, including high-entropy engineering [17], elemental doping, and the substitution and arrangement of components [18–20]. However, these studies necessitate intricate experimental procedures and comprehensive characterization to elucidate the performance dynamics induced by variations in composition or structure [21].

Recently, machine learning (ML) has gained extensive traction in the development of key materials [22,23]. It has been widely applied to design top-performing alloys, including copper alloys [24–26], magnesium alloys [27,28], electrostatic polymers [29], and shape memory alloys [30,31]. Moreover, ML methods are utilized to predict density functional theory (DFT) energies and forces at a notably reduced cost [32]. In the realm of superconducting materials, ML models not only accurately forecast superconducting transition temperatures but also effectively differentiate between superconductors and nonsuperconductors [33]. In the field of catalysis, ML is employed to predict performance and to screen catalyst formulations [34–38]. In the battery sector, researchers have developed ML models that facilitate and expedite the design of high-performance electrolytes [39]. To direct the synthesis of highly conductive solid electrolytes, an ML framework has been created that correlates microscopic morphology images with ionic conductivities [40]. Although ML has shown great promise in material development, its success is largely dependent on the careful selection and transformation of input features, a critical process known as feature engineering, which ensures the accuracy and predictive power of models.

From a data standpoint, we have assembled an extensive array of datasets to cultivate supervised ML models capable of predicting and optimizing the energy density delivered by various cathode materials. Given the intricate relationship between material properties and energy density, data-driven methodologies are especially apt for this investigation. We have compiled 73 data points from 51 published articles and utilized the elemental composition of NASICON cathode materials as the predictive features in our model. To enhance the precision of our predictions, we trained and evaluated a suite of ML models, including attention-Bayesian neural networks (AttenBNN), support vector machine (SVM), random forest (RF), and the K-nearest neighbor (KNN) algorithm. Our findings indicate that higher entropy and lower electronegativity are advantageous in enhancing energy density. Guided by this model, we engineered a cathode material, Na_3_Mn_0.5_V_0.5_Ti_0.5_Zr_0.5_(PO_4_)_3_ (NMVTZP), with an impressive energy density of 465 Wh kg^−1^, thereby validating the substantial potential of data-driven strategies in the advancement of materials development.

## Results and Discussion

Based on relevant researches of NASICON cathodes, we applied ML approach to explore effective NASICON cathode optimization strategies. By replacing transition metal ions in the Na_3_M_2_(PO_4_)_3_ framework with other elements, these strategies are prone to enhance the electronic structure of the material, optimize the sodium ion diffusion channels, and stimulate additional redox reactions, thus improving the performance of NASICON. Substitution with elements like Fe, Mn, Cr, and Ti has been widely used to activate higher-voltage redox couples, improve capacity, and stabilize phase transitions. For example, rational Al^3+^ and Cr^3+^ doping is prone to stimulate the additional V^4+^/V^5+^ redox couple and Mn^3+^/Mn^4+^ redox couple, and help improve Na^+^ mobility and suppress degradation. Ti species are beneficial to enhance the cyclic performance of cathodes and arouse the Mn^3+^/Mn^4+^ redox reaction in Mn-based NASICON cathodes [41]. Zr species can decrease the sodium-ion diffusion barriers, thus optimizing electrochemical performance of cathodes [42]. Y doping is beneficial to improving the enhanced intrinsic electronic conductivity and Na ion mobility [20]. Some high-valent ions such as Sn^4+^ [43] and Mo^6+^ [44] offer additional advantages. These ions are typically electrochemically inactive but contribute to structural reinforcement, improved Na^+^ diffusion channels, and enhanced thermal stability.

**Fig. 1.**
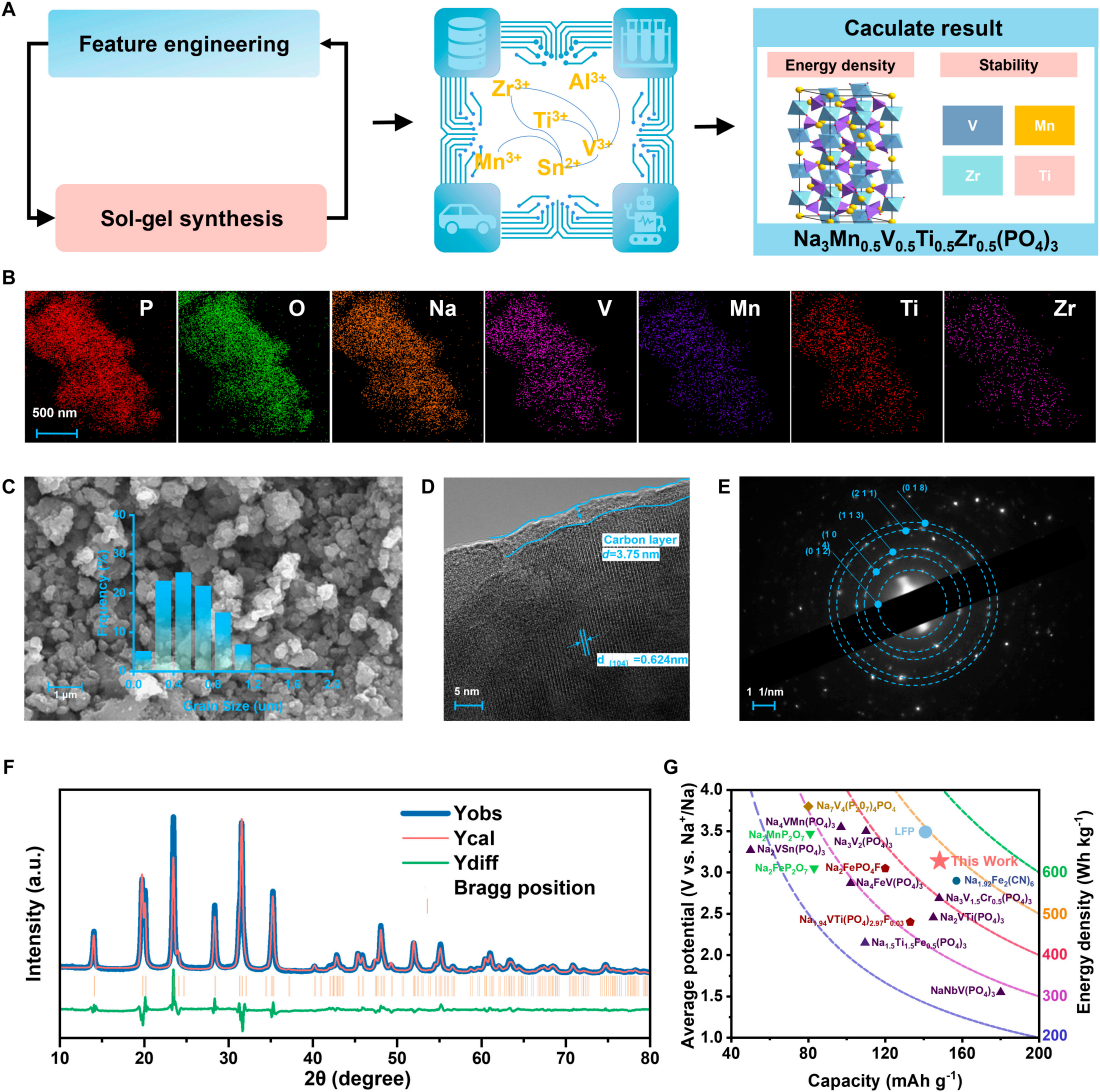
(A) Material selection method integrated with ML. (B) HAADF STEM image and corresponding EDS mapping results for designed NMVTZP materials. (C) SEM images (the inset is the particle size distribution curve of as-prepared NMVTZP). (D) HRTEM images. (E) SAED pattern. (F) XRD pattern and Rietveld refinement result of the NMVTZP. (G) Comparison of energy density with other materials.

Initially, we selected several cohorts of materials that exhibit high energy density and robust stability by means of data anXalysis. These selected materials were subsequently synthesized via the sol–gel method, which facilitates atomic-level mixing. Following a comparative analysis of material performance, NMVTZP was identified as the target cathode material due to its optimal balance of voltage plateau, energy density, and cycle stability (Fig. 1A). This material is poised for application in large-scale energy storage systems, including those supporting wind power and photovoltaic installations, as well as grid energy storage and EVs.

We delved deeper into the study of NMVTZP materials. Within the NASICON framework, the Mn, V, Ti, and Zr atoms occupy the octahedral positions of 12c in the crystal lattice. The TMO_6_ (where TM represents Mn, V, Ti, or Zr) octahedra and PO_4_ tetrahedra are interconnected through shared oxygen atoms, constructing a resilient 3-dimensional framework structure (Fig. 1A). The Na1 ions are located at the Wyckoff position of 6B, surrounded by a hexacoordinate oxygen environment, while the Na2 ions are positioned at 18e, encapsulated within an octacoordinate oxygen environment.

The elemental ratios were quantified using energy-dispersive spectroscopy (EDS) analysis. The obtained data exhibit a high degree of conformity with the initially prescribed composition (Table S1). High-angle annular dark-field scanning transmission electron microscopy (HAADF-STEM) images, along with the corresponding EDS elemental mappings (Fig. 1B), reveal an even distribution of Mn, V, Ti, and Zr throughout the NMVTZP material at the observed resolution. Transmission electron microscopy (TEM) and scanning electron microscopy (SEM) were employed to investigate the morphology and crystallographic features of NMVTZP. As depicted in Fig. 1C, the NMVTZP particles assume an irregular polyhedral morphology with sizes varying between 0.15 and 1.49 μm. The particle size distribution of NFMVTAP [Na_3.4_Fe_0.4_Mn_0.4_V_0.4_Ti_0.4_Al_0.4_(PO_4_)_3_] is similar to that of NMVTZP, indicating their similarity in preparation methods and material characteristics (Fig. S1). Figure 1D clearly shows the crystalline structure of NMVTZP along with an amorphous carbon coating. The high-resolution TEM (HRTEM) image of NMVTZP reveals distinct lattice fringes with an interplanar spacing of 0.624 nm, which corresponds to the ($\bar{1}12$) plane of the NASICON framework. The carbon layer, approximately 3.75 nm in thickness, greatly enhances the conductivity of the NASICON matrix, thereby improving the reaction kinetics. The selected-area electron diffraction (SAED) pattern in Fig. 1E clearly resolves lattice planes corresponding to (012), (104), (113), (211), and (018). The NFMVTAP synthesized via the same method also presents similar morphology and particle size distribution (Fig. S2).

Rietveld refinement analysis of the x-ray diffraction (XRD) data was performed to examine the crystal structure and purity of the sample (Fig. 1F). In line with the standard CIF (crystallographic information file) card for Na_3_V_2_(PO_4_)_3_, the NMVTZP sample exhibits a single, pure phase that conforms to the characteristic rhombohedral NASICON structure of the R-3c space group, with no detectable impurity phases. As detailed in Table S2, the Rietveld refinement yields convergence values of *Rwp* = 8.62% and *Rp* = 10.8%, based on the XRD data, with plausible displacement and site occupation factors for all constituent atoms. The crystallographic parameters of NMVTZP are established as *a* = *b* = 8.807731 Å and *c* = 22.168936 Å, with a *lattice volume* of 1,489.33 Å^3^. As recently reported, a larger lattice structure is indicative of more rapid Na^+^ ion migration and a more stable framework, which accounts for its enhanced rate performance [45]. For several other materials synthesized, XRD tests were also carried out and the results showed that all materials possess high crystallinity and all diffraction peaks also correspond well to Na_3_V_2_(PO_4_)_3_ phase (Figs. S3 to S6).

Figure 1G encapsulates the discharge voltage profiles plotted against specific capacity for the current polyanionic material cathodes. For superior energy density performance, both elevated discharge voltage and increased specific capacity are paramount. In this study, it is noteworthy that NMVTZP attains an impressive energy density of 465 Wh kg^−1^, which is achieved through a specific capacity of 148.27 mAh g^−1^ at an average voltage of 3.14 V. This outcome suggests that SIBs have the potential to rival the position of LiFePO_4_ (LFP) in energy storage applications.

### Data acquisition and preprocessing

**Fig. 2.**
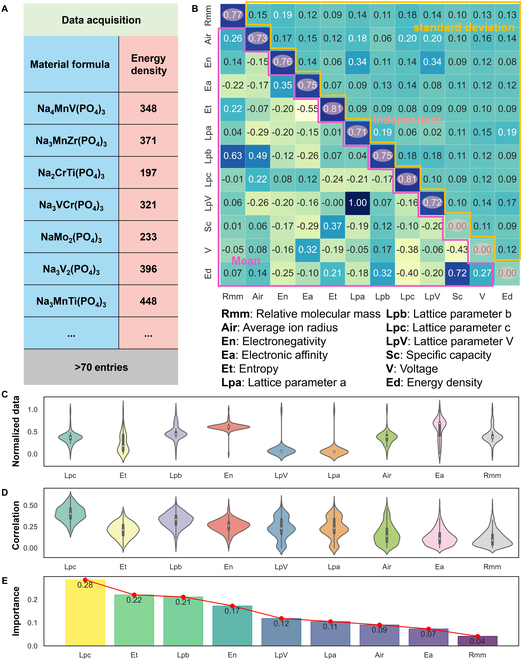
(A) Data acquisition process. (B) The correlation coefficient between the features: The lower triangle is the mean of the correlation coefficient, the upper triangle is the SD (standard deviation) of the correlation coefficient, and the diagonal is the independent coefficient. (C) Normalized data distribution. (D) Correlation coefficient distribution between features and energy density. (E) Importance of different features.

High-quality and reliable data are crucial to data-driven research. The compound Na*_x_*(M)*_y_*(PO_4_)_3_, where M denotes transition metal elements, has been the subject of extensive study as a cathode material for SIBs. As illustrated in Fig. 2A, we have compiled a dataset comprising 73 data points, which range in energy densities from 197 to 470 Wh kg^−1^. The complete dataset and corresponding references are provided in Supplementary Dataset.

The chemical formula of the materials serves as the primary input. Subsequently, the material is characterized through ML to optimize its representation for ML algorithms. The process involves selecting initial input features such as relative molecular mass, electronegativity of the transition metal elements, electron affinity of the transition metal elements, entropy value, and lattice parameters. The weighted mean method is employed to compute the equivalent electronegativity and electron affinity. The output is defined by the average discharge voltage, specific discharge capacity, and energy density. Entropy is a concept in statistical thermodynamics that describes the degree of chaos in a system, and its formula is as follows [46]:

$$S=-R\sum_{i=1}^{n}x_{i}\text{ln}x_{i}$$ (1)

where R is the ideal gas constant and $x_{i}$ represents the molar fraction of the *i* component. For a good number of components *n*, the configuration entropy reaches the largest value when the atomic fraction is the same for all components (equimolarity). Descriptions and calculations for additional input feature parameters (*relative molecular mass*, *average ion radius*, *electronegativity*, *electronic affinity*, *lattice parameter a*, *lattice parameter b*, *lattice parameter c*, *lattice parameter V*) are detailed in the Supplementary Materials. Utilizing the linear correlations between variables, the preliminary input features are initially screened. The feature correlation is random, which is primarily determined by the calculated dataset, and its representativeness is poor (Fig. S7).

We employ an autoencoder-based dimensionality reduction from 9 input variables to a 4-dimensional latent space, coupled with t-SNE (t-distributed stochastic neighbor embedding) visualization of reconstructed features, to systematically evaluate variable interdependencies and data reconstructibility within the NASICON cathode dataset. The analysis reveals strong feature independence among the majority of parameters—such as electronegativity, configurational entropy, and lattice parameters—indicating minimal informational overlap and validating their collective inclusion in predictive modeling (Fig. S8).

Thus, the bootstrap random sampling method was used, where 50 samples were selected each time, and the sampling was repeated 300 times. The mean and SD plots of the correlation for each variable were then generated (Fig. 2B and D). It can be observed that the mean correlation between the 4 features (lattice parameter c, lattice parameter b, electronegativity, and entropy) and energy density is high, with a small SD, indicating that the correlation between the 4 features is reliable. As shown in Fig. 2C, the normalized data distributions of lattice parameter b, lattice parameter c, and Electronegativity follow a Gaussian distribution, while the distribution of Entropy is more uniform. We believe that entropy has a more effective predictive ability at both high and low energy densities. Therefore, it is also necessary to consider the independence and distribution of the features themselves using the Gini coefficient.

Based on the above research, we ranked the importance of the 4 features as follows:

$$I=\rho \cdot \left(1-\sigma \right)\cdot \alpha \cdot \left(1-\sigma \right)\cdot \left(1+G\right)$$ (2)

where I is the importance of the 4 features, ρ is the mean of the correlation coefficient, σ is the SD of the correlation coefficient, α is the independence coefficient, and G represents the Gini coefficient. Finally, *lattice parameter c* is established as the most important feature (Fig. 2E).

**Fig. 3.**
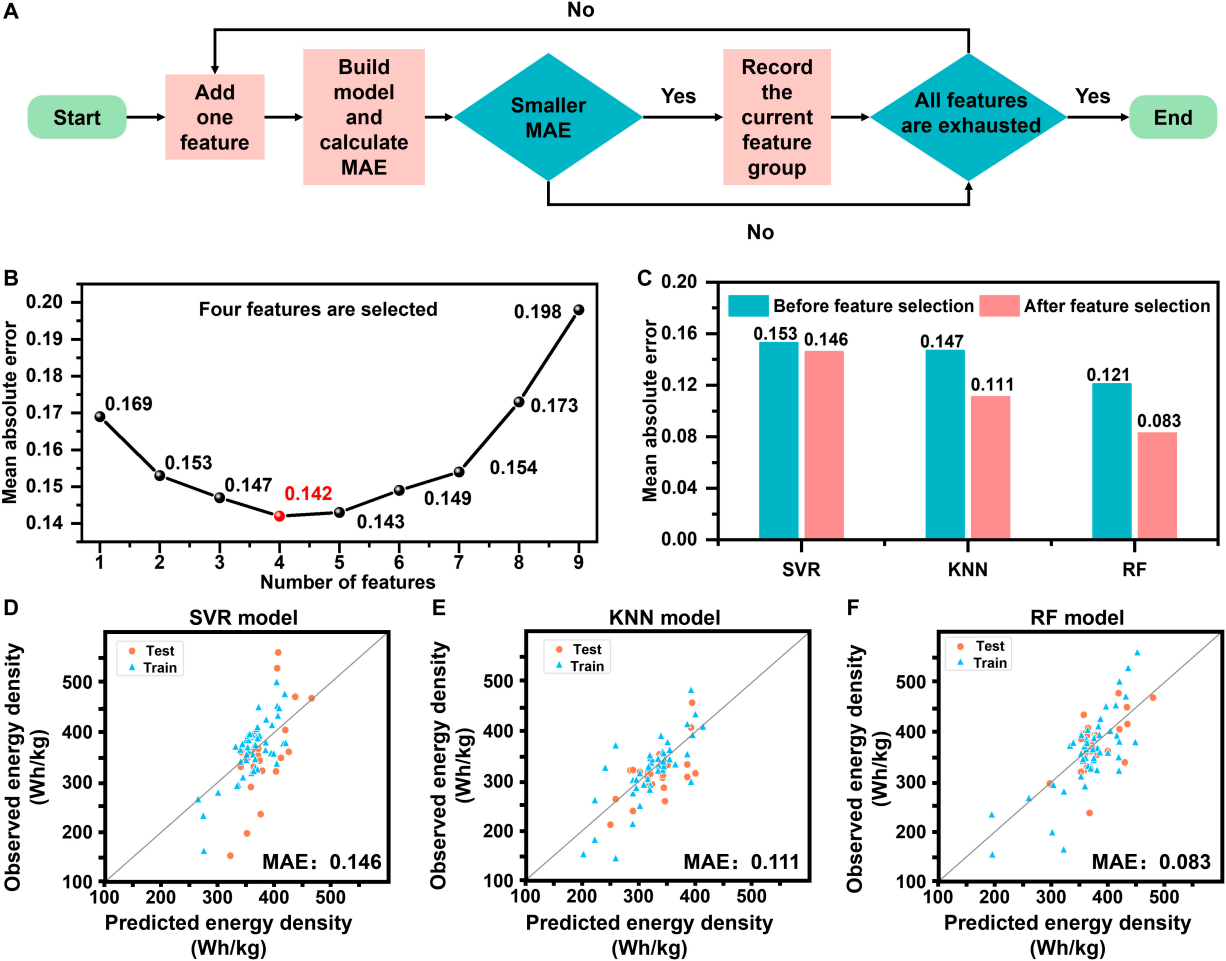
(A) Forward feature selection method. (B) Feature forward selection results. (C) Comparison of errors before and after feature selection. (D to F) Plots of predicted energy density versus observed energy density for the 4-feature SVR (support vector regression), KNN, and RF model, respectively.

To determine the number of input features for model prediction, we employed the forward feature selection method. The specific procedure is as follows: Initially, each feature is individually selected as the input, the model is trained, and the average mean square error between the predicted and actual values is computed. By comparison, the optimal feature is identified when only one input feature is used. This process is repeated, incrementally increasing the number of features, and the optimal feature set for each feature count is determined (Fig. 3A) [47]. Ultimately, we conclude that 4 features are sufficient to achieve the minimum error (Fig. 3B).

To assess the accuracy of feature selection, we selected RF, SVM, and the KNN algorithm for evaluation. Fifty datasets were employed as the training set for the model, while the remaining 26 datasets served as the test set. Hyperparameter optimization was conducted using Grid Search CV.

As depicted in Fig. 3C, the mean absolute error (MAE) values for all algorithm test sets decreased when utilizing the refined features to predict energy density, indicating that the predictive performance of the model has been enhanced through the forward selection of features. Grid Search CV is an ML technique for parameter tuning that identifies the optimal model parameters by iterating over a defined range of parameter combinations and employing cross-validation for each set. Specifically, Grid Search CV defines a grid of parameters, encompassing those to be tuned and their potential values [48]. It then trains and evaluates the model using various combinations of these parameters, ultimately selecting the combination that yields the best performance as the optimal model parameters. The workflow for Grid Search CV is detailed in the Supplementary Materials. The primary benefit of Grid Search CV is its systematic exploration of the parameter space to pinpoint the optimal parameter combination, thereby enhancing model performance and generalization capability. However, due to the iteration over all possible parameter combinations, the computational expense of Grid Search CV can be crucial. In the context of this small-sample regression problem, the computational cost is minimal and thus considered negligible. The results demonstrate that the predictive capabilities of ML are effectively enhanced through hyperparameter optimization and feature selection.

Based on the comparison of MAE values across all algorithms, the RF algorithm emerged as the most effective for prediction purposes. Figure 3F displays the scatterplot of predicted versus actual values for both the training and test sets using the RF algorithm. In contrast to Fig. 3D and E, the scatter distribution in Fig. 3F for the RF algorithm appears more tightly clustered.

Moreover, the predictability of any model for medium energy density is more accurate, and the prediction ability of high and low energy density samples is poor. This is because the total number of samples is small, especially the high and low energy density samples, which leads to inaccurate prediction results of the regression model (Fig. S9). We propose the AttenBNN model, which combines attention method and Bayesian neural networks (BNNs) to describe the uncertainty (Fig. S10).

Within the feature attention layer, an attention score is calculated for each input feature, signifying the feature’s importance in energy density prediction. These scores are then normalized via the Softmax function to produce weights for each feature, which determines the relative importance of each feature in the model’s prediction. Subsequently, a weighted sum operation is performed on the input features based on these normalized weights, yielding a weighted feature vector that integrates the importance of each feature and emphasizes those critical for energy density prediction. This weighted vector is supplied as input to subsequent model components, BNNs, for further energy density prediction tasks. The final output feature attention values are largely in line with the previously calculated importance. The specific values are as follows: *electronegativity*: 0.479, entropy: 0.764, *lattice parameter b*: 0.679, *lattice parameter c*: 0.722.

BNNs present a departure from traditional deterministic neural networks by adopting a probabilistic approach to modeling. In deterministic neural networks, parameters such as *weights* (*W*) are treated as fixed values, learned through optimization techniques like gradient descent. However, in BNNs, these parameters are regarded as random variables governed by probability distributions, allowing for the representation of uncertainty in model predictions. For better performance, we use the Monte Carlo dropout (MC-dropout) method to simulate BNNs. By applying dropout at test time and running multiple forward passes with different dropout masks, the model produces a distribution of predictions rather than a single point estimate. This distribution provides insights into the model uncertainty about its predictions, effectively regularizing the network. The dropout selection is 0.2. As shown in Figs. S11 and S12, the horizontal axis represents the actual energy density, and the vertical axis denotes the predicted energy density error with upper and lower bounds (1 SD), where the size of the scatter corresponds to the error magnitude. The upper and lower bounds of the error can completely cover the true value, verifying the feasibility of the model in material selection. However, it is also noteworthy that in the test set (Fig. S11b), the uncertainty intervals are relatively wide, and several predicted values exhibit considerable deviations from the actual values. This indicates that while the AttenBNN model effectively quantifies uncertainties in small datasets, its generalization ability and prediction accuracy for unseen data under small-sample conditions still need improvement. Future work will focus on expanding the dataset and optimizing the model structure to enhance its performance in practical applications.

### Model validation

Through analysis of the results above, we have identified the following insights: The lattice parameters notably impact energy density, with larger crystals tending to result in lower energy densities. We hypothesize that this may be attributed to the more intricate interactions among atoms in larger crystal structures. The entropy value has a pronounced effect on both capacity and energy density, with these 2 metrics showing a positive correlation. However, an increase in entropy often leads to a decrease in the average discharge voltage. Thus, it is essential to judiciously enhance the entropy value, in alignment with current mainstream research trends. Equivalent electronegativity is inversely related to energy density.

In SIB systems, entropy regulation plays a crucial role in determining the energy density of cathode materials, particularly through its influence on the interactions among constituent elements. In NASICON-type frameworks, an increase in configurational entropy introduced through multi-element substitution on the transition metal site can lead to more complex interatomic interactions. This complexity has been shown to stabilize lattice structure and, in some cases, activate additional redox couples (such as Mn^3+^/Mn^4+^ and V^4+^/V^5+^), thereby enhancing the overall energy density of cathode [45,49].

The electronegativity difference between transition metal ions and anions or anionic ligands plays a critical role in determining the nature of the resulting chemical bonds. A larger electronegativity difference generally leads to the formation of more ionic bonds, while smaller differences favor covalent bonding. Materials characterized by predominantly ionic bonding tend to exhibit densely packed structures, whereas those with more covalent character typically adopt more loosely packed configurations. The degree of structural packing not only influences the phase or crystal stability of the material but also impacts the specific site energy of ions, which is closely associated with the material’s electrochemical potential [50].

The lattice parameters exert a profound influence on the energy density of SIB cathode materials [51]. When the lattice parameters are within an appropriate range, the crystal structure of the cathode material achieves enhanced stability. This stability enables the material to preserve its structural integrity more effectively during the charge and discharge processes, thereby minimizing capacity loss and contributing to the maintenance of a relatively high energy density. Simultaneously, the suitable lattice parameters facilitate smoother sodium-ion diffusion channels, which not only reduces the diffusion barrier for sodium ions but also enhances the rate performance of the battery, further supporting the improvement of energy density. Moreover, appropriate lattice parameters can provide a greater number of sodium-ion storage sites, allowing for increased participation of sodium ions in electrochemical reactions and thus boosting the specific capacity of the material, which is directly beneficial to the elevation of the battery’s energy density. Conversely, if the lattice parameters deviate from the optimal range, it may lead to internal stress within the crystal structure, hinder sodium-ion diffusion, and reduce the specific capacity, all of which can adversely affect the energy density of the SIBs. In summary, the lattice parameters influence the energy density of SIB cathode materials by simultaneously affecting structural stability, sodium-ion diffusion kinetics, and the specific capacity of the material.

As shown in Fig. S13, the high-performance NASICON cathode needs to meet:

1. *Entropy* > 0.5 (activation of multi-electron reaction)
2. *Equivalent electronegativity* < 3.4 (enhanced ionic bond stability)
3. *Latice parameter c*: 21.4 to 22.2 Å (balance ion diffusion and structural integrity)

Consequently, to enhance the energy density of cathode materials, it is imperative to design and develop NASICON materials characterized by smaller crystals, higher entropy values, and lower equivalent electronegativity. Drawing on the electronegativity and ionic radius of various elements, we selected those with marked differences, such as V^3+^, Mn^2+^, Al^3+^, Mo^3+^, Sc^3+^, Fe^3+^, Ti^3+^, and Zr^4+^. The parameters of these elements are detailed in Table S3. To validate our findings, we created the following combinations of elements: (Mn, V), (Mn, V, Al), (Mn, V, Mo), (Mn, V, Sc), (Mn, V, Ti, Zr), and (Mn, V, Fe, Ti, Al). The property parameters of these materials are presented in Table S4, such as Na_4_MnV(PO_4_)_3_ (NMVP), Na_11/3_Mn_2/3_V_2/3_Al_2/3_(PO_4_)_3_ (NMVAP), Na_11/3_Mn_2/3_V_2/3_Mo_2/3_(PO_4_)_3_ (NMVMP), Na_11/3_Mn_2/3_V2/3Sc_2/3_(PO_4_)_3_ (NMVSP), NMVTZP, and Na_3.4_Fe_0.4_Mn0.4V_0.4_Ti_0.4_Al_0.4_(PO_4_)_3_ (NMVFTAP). Unfortunately, part of synthesis attempts was unsuccessful, including Na_3_Mn_2/3_V_2/3_Sn_2/3_(PO4)_3_, Na_3.5_Mn_0.5_V_0.5_Ni_0.5_Co_0.5_(PO_4_)_3_, Na_3_Mn_0.5_V_0.5_Sc_0.5_Zr_0.5_(PO_4_)_3_, Na_3.4_Fe_0.4_Mn_0.4_V_0.4_Ti_0.4_Mo_0.4_(PO_4_)_3_, and Na_3.4_Fe_0.4_Mn_0.4_V_0.4_Ti_0.4_Y_0.4_(PO_4_)_3_, using the sol–gel method under the same experimental conditions. The failed synthesis of NVMSP [Na_11/3_Mn_2/3_V_2/3_Sc_2/3_(PO_4_)_3_] and NFMVTYP [Na_3.4_Fe_0.4_Mn_0.4_V_0.4_Ti_0.4_Y_0.4_(PO_4_)_3_] is ascribed to unexpected impurity. Bare Na_4_VMn(PO_4_)_3_ exhibits NASICON R-3c structure, while unexpected impurities such as Na_4_SnO_4_ gradually appear with the increase of Sn content [43]. Yttrium substituted NASICON is faced with similar dilemma. When yttrium content exceeds a certain limit, some impurities will occur, such as YPO_4_ or Na_3_Y_2_(PO_4_)_3_ [52].

**Fig. 4.**
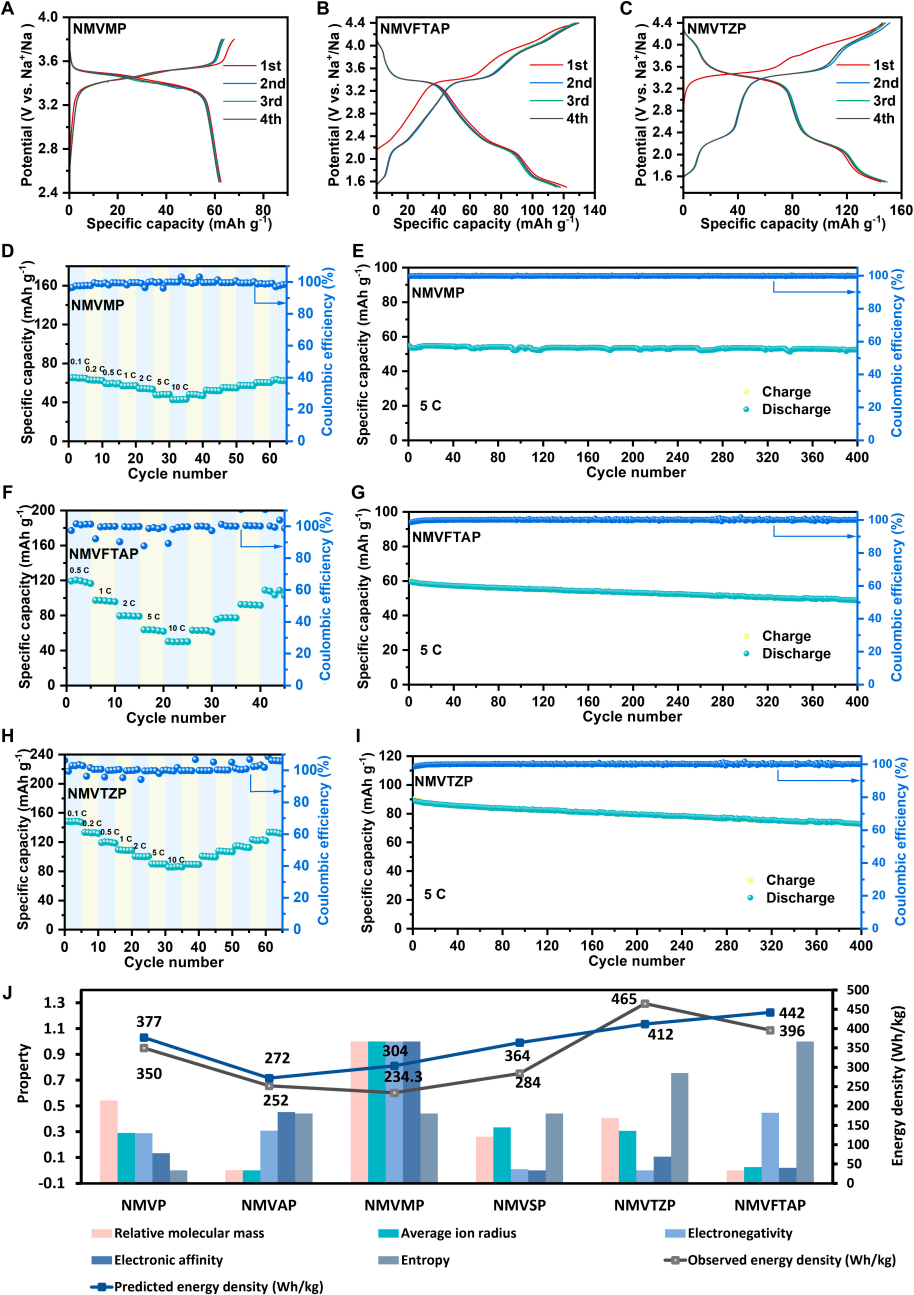
Experimental results. (A to C) Charge–discharge curves for the first 4 cycles of NMVMP, NMVTZP, and NMVFTAP electrodes, respectively. (D to I) Rate performance from 0.1 to 10 C and cycling performance at 5 C for 400 cycles of NMVMP, NMVTZP, and NMVFTAP electrodes, respectively. (J) Physical and electrochemical properties of the 6 materials and their corresponding energy densities.

To assess the electrochemical performance of these cathode materials, 6 electrodes were fabricated into cell batteries, with metallic Na used as both the anode and reference electrode. As depicted in Fig. 4A to C and Figs. S14 to S16, at a discharge rate of 0.1 C, the specific capacities of the 6 materials were measured to be 107.82, 70.92, 67.92, 76.55, 148.27, and 129.38 mAh g^−1^, respectively. The corresponding energy densities were calculated to be 350.0, 252.0, 234.3, 284.0, 465.0, and 396.0 Wh kg^−1^. A comparison of the initial 4 cycles for these 6 materials revealed that, within a certain range, increasing the entropy value and decreasing the electronegativity can effectively enhance the energy density.

High-entropy materials typically possess a greater number of redox pairs, which introduces the potential for higher voltage plateaus. For instance, the capacity of NMVTZP is derived from the redox pairs of Mn^2+^/Mn^3+^/Mn^4+^, Ti^3+^/Ti^4+^, and V^2+^/V^3+^/V^4+^/V^5+^, while the capacity of NMVFTAP is sourced from the redox pairs of Mn^2+^/Mn^3+^/Mn^4+^, Ti^3+^/Ti^4+^, V^2+^/V^3+^/V^4+^/V^5+^, and Fe^2+^/Fe^3+^. Generally, in NASICON-type electrodes, high-voltage plateaus above 4.0 V are attributed to the redox pairs of Mn^3+^/Mn^4+^ (4.0 V) and V^4+^/V^5+^ (3.9 V), which are the focal points of NASICON cathode research. The addition of extra redox couples contributes to higher operating voltages and specific capacities, thereby improving energy density. Specifically, NMVTZP exhibits a more uniform and reversible charge/discharge profile, particularly at high voltages. During discharge, the NMVTZP electrode displays a distinct voltage plateau for V^4+^/V^5+^ and Mn^3+^/Mn^4+^ above 4.0 V, which is less pronounced in the NMVFTAP electrode, accounting for its superior energy density.

Rate performance is crucial for practical applications. As shown in Fig. 4D, F, and H, in contrast to other materials, the NMVTZP electrode demonstrated exceptional rate capabilities. At discharge rates of 0.1, 0.2, 0.5, 1, 2, 5, and 10 C, the NMVTZP electrode delivered capacities of 148.3, 133.8, 119.3, 109.5, 100.5, 90.2, and 85.8 mAh g^−1^, respectively. Notably, at the high current rate of 5 C, the NMVTZP electrode retained the highest capacity compared to the other electrodes. When the rate was reduced back to 0.1 C, a capacity of 133.3 mAh g^−1^ was recovered, indicating excellent rate performance. The cycling stability of each material at a 5-C rate was also evaluated (Fig. 4E, G, and I). The NMVTZP material retained 78.1% of its initial capacity after 400 cycles of high-rate cycling, signifying a highly reversible Na^+^ storage process during charge and discharge. As illustrated in Fig. 4J, the energy density prediction model effectively uses available information to infer the potential energy density of materials and quickly identifies promising new materials within the complex material group. DFT, based on quantum mechanics, accurately calculates material properties from the electronic structure. It applies to various material systems, including crystals, amorphous materials, surfaces, and interfaces, and provides detailed microscopic information like electronic structure and density of states. This aids in in-depth understanding of material properties. However, DFT calculations are computationally intensive and time consuming, especially for large-scale material screenings. For complex systems like strongly correlated ones, its accuracy may be limited, requiring advanced methods for correction. Moreover, the choice of exchange-correlation functionals and calculation parameters can affect results, demanding professional knowledge for proper selection [53–55]. Our machine learning approach, free from subjective selection and conventional knowledge constraints, swiftly obtains material energy density data. With experimental validation ensuring sufficient accuracy for material screening, it offers stronger practicality.

As detailed in Table S5, it is evident that our developed material demonstrates remarkable overall performance compared to current advanced materials. It shows excellent specific capacity, average voltage, and cycling performance. Some materials may have higher specific capacity but compromise on cycling performance and average voltage. Others may have a higher average voltage, yet their energy density is inferior to our material.

### Material characterization

**Fig. 5.**
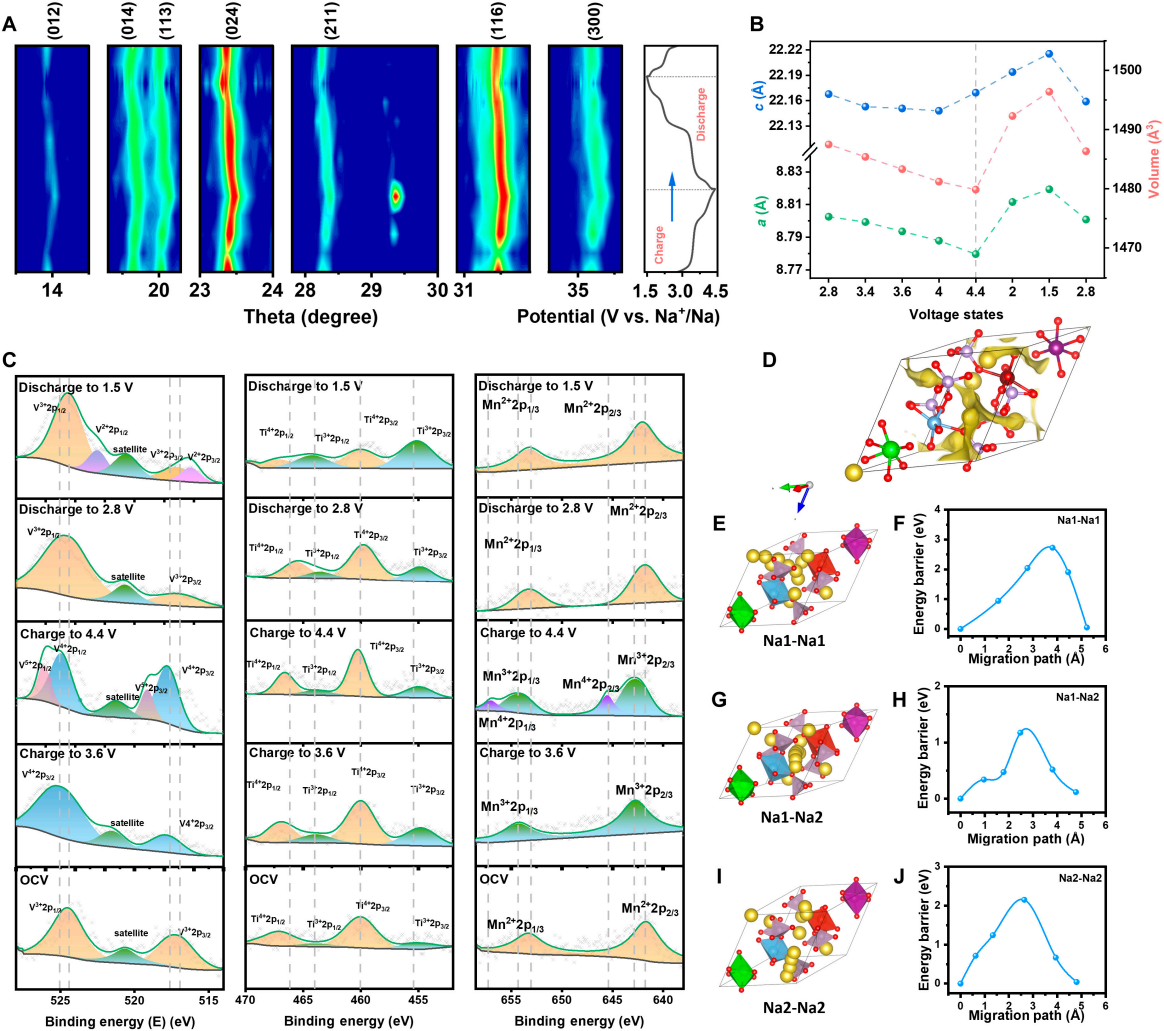
(A) XRD patterns at different electrochemical stages and the corresponding 2D contour plot of the NMVTZP. (B) Crystal parameters of NMVTZP under different voltage. (C) XPS spectra of V, Ti, and Mn that were collected from the OCV (open circuit voltage), charged to 3.6 V, charged to 4.4 V, discharged to 2.8 V, and discharged to 1.5 V of the NMVTZP. (D) Schematic illustration of the possible migration pathways represented by yellow in the primitive cell model of NMVTZP. (E, G, and I) Calculated Na^+^ diffusion pathways of (Na1 to Na1), (Na1 to Na2), and (Na2 to Na2) in NMVTZP material. (F, H, and J) Corresponding migration energy barriers within the different Na^+^ groups illustrated.

The sodium storage mechanism of NMVTZP was elucidated through in situ XRD analysis. Figure 5A depicts the distinctive diffraction peaks of NMVTZP at various charge/discharge stages, along with the corresponding 2D contour plots. A close examination of the patterns reveals that the diffraction peaks corresponding to (012), (014), (113), (024), (211), (116), and (300) shift progressively toward higher angles during the charging process, which is indicative of a contraction of the unit cell as Na^+^ ions are extracted. During the subsequent discharge, these diffraction peaks revert to their original positions, confirming a solid-solution-type sodium ion storage behavior [56]. The solid solution mechanism is known to entail a lower migration barrier for Na^+^ compared to the biphasic reaction mechanism, thereby enhancing the kinetic reaction rate due to the absence of ordering rearrangements of sodium ions and vacancies [56]. Additionally, it is noteworthy that the (211) diffraction peak intermittently emerges and vanishes in the voltage range of 3.8 to 4.4 V, suggesting a biphasic storage mechanism for sodium ions [41]. In summary, the predominant sodium storage mechanism in NMVTZP is a solid-solution reaction that coexists with a biphasic reaction.

To assess the diffusion capability of Na^+^, we employed the galvanostatic intermittent titration technique (GITT). The GITT curves and the calculated DNa^+^ values for the NMVTZP cathode during the second cycle are presented in Figs. S17 to S22. The NMVTZP exhibits minor fluctuations in DNa^+^ values within a relatively narrow range of approximately 10^−10^ to 10^−13^ cm^2^ s^−1^ during cycling at room temperature. Specifically, a decrease in DNa^+^ is observed within the regions of the 4 redox plateaus, indicating slower dynamic processes.

To elucidate the charge compensation mechanism of Mn, V, Ti, and Zr in NMVTZP, ex situ XPS (x-ray photoelectron spectroscopy) analysis was conducted across a voltage range from 1.5 to 4.4 V (Fig. 5B). For the vanadium species, as the voltage increased, all V3^+^ ions were completely oxidized to V4^+^ by 3.6 V. At 4.4 V, the emergence of V5^+^ peaks indicated that some V4^+^ ions were further oxidized to V5^+^. Upon discharging to 1.5 V, the appearance of V2^+^ peaks in the spectrum was observed. In the case of titanium, 2 valence states were detected during the experiment. As charging progressed, the concentration of Ti4^+^ ions increased, while Ti2^+^ ions decreased, suggesting an active role for titanium in the redox process. Manganese behavior during charging was analogous to vanadium, with Mn2^+^ ions progressively oxidizing to Mn3^+^ and then to Mn4^+^. During discharge, all manganese ions reverted to their initial Mn2^+^ state. As depicted in Fig. S23, the valence state of V4^+^ remained unchanged during the charge and discharge processes, indicating the absence of redox reactions involving vanadium.

Bond valence (BV) analysis can visually delineate ion transport pathways and effectively confirm the ion transport mechanism. Utilizing bond valence energy landscape (BVEL) analysis, the potential Na^+^ migration pathways in NMVTZP were identified. As shown in Fig. 5C, NMVTZP features expanded Na^+^ migration channels around the TMO_6_ (TM = Mn, V, Ti, Zr) octahedra compared to NVP [Na_3_V_2_(PO_4_)_3_], which enhanced Na^+^ diffusion kinetics due to the multi-element substitution [57].

To gain insight into the specific migration behavior of sodium ions within the crystal lattice, DFT calculations were performed. Based on the simulated Na^+^ migration paths, 3 plausible migration pathways were constructed: Na1–Na1 (P1), Na1–Na2 (P2), and Na2–Na2 (P3). Employing the climbing image nudged elastic band (CI-NEB) method, the migration energy barriers for paths P1, P2, and P3 were calculated to be 2.73, 1.17, and 2.15 eV, respectively (Fig. 5D to I). Consequently, the most favorable diffusion pathway for Na^+^ in the NMVTZP structure is the Na1–Na2 mode. The low diffusion energy barrier is a key factor contributing to the exceptional rate performance of this material.

## Conclusion

This report delineates a data-driven methodology for the optimization of cathode materials. Drawing upon data from the scientific literature and experimental findings, we developed an ML model that revealed elemental electronegativity and material entropy to be pivotal factors influencing energy density. Leveraging this insight as a design paradigm, we have introduced a suite of cathode materials characterized by high entropy and low electronegativity, capable of achieving an energy density of 465 Wh kg^−1^. Our models and the materials developed underscore the substantial influence of elemental properties on the overall energy density of materials. In the future, we plan to incorporate more SIB cathode materials of various structural types and introduce ensemble learning to enhance prediction accuracy. We contend that this research showcases the potential of data-driven strategies in constructing predictive models and uncovering novel insights in material science.

## Materials and Methods

Details about the methods used in this work are available in the Supplementary Materials.

## Data Availability

All data are available in the main text or the Supplementary Materials. Source data are available from the corresponding author upon reasonable request.

## Supplementary Materials

Supplementary 1 Supplementary Text Figs. S1 to S23 Tables S1 to S5 Data file S1 References [46,49,58–62]
