# Supplementary material for: Machine Learning for Selecting High-Energy Phosphate Cathode Materials
Source: Research (Wash D C). 2025 Jul 29;8:0794. doi: 10.34133/research.0794 (PMC12304885; doi:10.34133/research.0794)
Supplement: Supplementary 1 — Supplementary Text Figs. S1 to S23 Tables S1 to S5 Data file S1 References [46,49,58–62] [file research.0794.f1.zip › Revised Supplementary Information without Mark.docx]

Supporting Information

**Machine Learning for Selecting High-Energy Phosphate Cathode Materials**

Yongchun Dang, ^a^ Zechen Li, ^b^ Yongchao Yu, ^a^ Xiwei Bai, ^c^ Li Wang, ^d^ Xuelei Wang, ^c^ Peng Liu, ^a,e^ Chen Sun, ^b^ Xunli Zhou, ^e^ Zhenpo Wang,*^a^ Yongjie Zhao,*^b^ Xiangming He *^d^ and Lei Li *^a,e^

*a. National Engineering Research Center of Electric Vehicles, Beijing Co-innovation Centre for Electric Vehicles, Beijing Institute of Technology, Beijing 100081, China. E-mail: wangzhenpo@bit.edu.cn, flytolilei@bit.edu.cn*

*b. Beijing Key Laboratory of Construction Tailorable Advanced Functional Materials and Green Applications, School of Materials Science and Engineering, Beijing Institute of Technology, Beijing 100081, China. E-mail: zhaoyj14@bit.edu.cn*

*c. Institute of Automation, Chinese Academy of Sciences, Beijing 100190, China.*

*d. Institute of Nuclear and New Energy Technology, Tsinghua University, Beijing 100084, China. E-mail:* [*hexm@tsinghua.edu.cn*](mailto:hexm@tsinghua.edu.cn)

*e. Beijing Institute of Technology Chongqing Innovation Center, Chongqing 401120, China.*

**1. Features calculation**

According to the molecular formula of the active material, its relative molecular mass can be easily calculated, and for its average ionic radius, the equation is calculated as follows**:**

$$\dot{\boldsymbol{r}}\boldsymbol{=}\frac{\sum_{\boldsymbol{i=1}}^{\boldsymbol{n}} \boldsymbol{r}_{\boldsymbol{i}}}{\boldsymbol{n}}$$

where $r_{i}$ represents the ionic radius of the $i$ transition metal ions.

For its electronegativity or electronic affinity, the equation has a similar form**:**

$$\dot{\boldsymbol{E}}\boldsymbol{=}\sum_{\boldsymbol{i=1}}^{\boldsymbol{n}} \boldsymbol{e}_{\boldsymbol{i}}$$

where $e_{i}$ represents the electronegativity or electronic affinity of the $i$ transition metal ions.

As for the lattice parameter a, b, c and V which describe the crystal size, are all from published data.

**2. The workflow of Grid Search CV**

Specify a parameter grid: Define the parameters to be tuned and their value ranges. For example, you can set different values for the depth of the decision tree model, and different values for the C and gamma parameters of the SVM model.

Cross-validation: Divide the dataset into subsets, and then use one of the subsets as the validation set and the remaining subsets as the training set for each training session. Cross-validation allows for a more accurate assessment of model performance and reduces bias caused by different sample divisions. Grid Search CV typically uses k-fold cross-validation, where k is a pre-set integer, and k = 5 is used in this study.

Training and evaluation: For each set of parameter combinations, the model was trained on the training set and the model performance was evaluated on the validation set, and the evaluation metric used in this study was RMSE.

Model selection: Based on the results of cross-validation, identify the best-performing combination of parameters and use those parameters to train the entire training set. The resulting model is the best model selected by Grid Search CV.

For the KNN model, the main hyperparameters include n_neighbors, p, and weights. Using the grid search algorithm, the optimal parameter values were determined to be n_neighbors=2, p=2, and weights='uniform'.

| **def** my_knn(feature_train, target_train):  kn = KNeighborsRegressor()   kn_param_grid1 = {**'n_neighbors'**: range(1, 9)}  kn_best = GridSearchCV(kn, param_grid=kn_param_grid1, cv=5, scoring=**"neg_root_mean_squared_error"**, n_jobs=-1,  verbose=0)  kn_best.fit(feature_train, target_train)   kn_param_grid2 = {**'p'**: range(1, 6)}  kn_best2 = GridSearchCV(kn_best.best_estimator_, param_grid=kn_param_grid2, cv=5,  scoring=**"neg_root_mean_squared_error"**,  n_jobs=-1,  verbose=0)  kn_best2.fit(feature_train, target_train)   kn_param_grid3 = {**'weights'**: [**'uniform'**, **'distance'**]}  kn_best3 = GridSearchCV(kn_best2.best_estimator_, param_grid=kn_param_grid3, cv=5,  scoring=**"neg_root_mean_squared_error"**,  n_jobs=-1,  verbose=0)  kn_best3.fit(feature_train, target_train)   **return** kn_best3, kn_best3.best_estimator_ |
| --- |

For the RF model, the main hyperparameters include n_estimators, min_samples_split, max_features, max_depth and max_leaf_nodes. Using the grid search algorithm, the optimal parameter values were determined to be n_estimators=500, min_samples_split=7, max_features='sqrt', max_depth=100 and max_leaf_nodes=500.

| **def** my_randomforest(feature_train, target_train):  rf = RandomForestRegressor()   rf_param_grid1 = {**'n_estimators'**: [300, 500, 800, 1200, 2000, 3000]}  rf_best = GridSearchCV(rf, param_grid=rf_param_grid1, cv=5, scoring=**"neg_root_mean_squared_error"**, n_jobs=-1,  verbose=0)  rf_best.fit(feature_train, target_train)   rf_param_grid2 = {**'min_samples_split'**: [2, 5, 7, 8, 10, 20]}  rf_best2 = GridSearchCV(rf_best.best_estimator_, param_grid=rf_param_grid2, cv=5,  scoring=**"neg_root_mean_squared_error"**,  n_jobs=-1,  verbose=0)  rf_best2.fit(feature_train, target_train)   rf_param_grid3 = {**'max_features'**: [**'log2'**, **'sqrt'**, **None**]}  rf_best3 = GridSearchCV(rf_best2.best_estimator_, param_grid=rf_param_grid3, cv=5,  scoring=**"neg_root_mean_squared_error"**,  n_jobs=-1,  verbose=0)  rf_best3.fit(feature_train, target_train)   rf_param_grid4 = {**'max_depth'**: [20, 50, 100, 500, **None**]}  rf_best4 = GridSearchCV(rf_best3.best_estimator_, param_grid=rf_param_grid4, cv=5,  scoring=**"neg_root_mean_squared_error"**,  n_jobs=-1,  verbose=0)  rf_best4.fit(feature_train, target_train)   rf_param_grid5 = {**'max_leaf_nodes'**: [5, 50, 500, 5000]}  rf_best5 = GridSearchCV(rf_best4.best_estimator_, param_grid=rf_param_grid5, cv=5,  scoring=**"neg_root_mean_squared_error"**,  n_jobs=-1,  verbose=0)  rf_best5.fit(feature_train, target_train)   **return** rf_best5, rf_best5.best_estimator_ |
| --- |

For the SVR model, the main hyperparameters include kernel, gamma, and C. Using the grid search algorithm, the optimal parameter values were determined to be kernel='rbf', gamma=1, C=10.

| **def** my_SVR(feature_train, target_train):  svr = KNeighborsRegressor()   svr_param_grid1 = {**'kernel'**: **['linear', 'poly', 'rbf', 'sigmoid']**}  svr_best = GridSearchCV(svr, param_grid=svr_param_grid1, cv=5, scoring=**"neg_root_mean_squared_error"**, n_jobs=-1,  verbose=0)  svr_best.fit(feature_train, target_train)   svr_param_grid2 = {**'gamma'**: [0.001, 0.01, 0.1, 1.0, 10.0, 100.0, 1000.0]}  svr_best2 = GridSearchCV(svr_best.best_estimator_, param_grid=svr_param_grid2, cv=5,  scoring=**"neg_root_mean_squared_error"**,  n_jobs=-1,  verbose=0)  svr_best2.fit(feature_train, target_train)   svr_param_grid3 = {**'C'**: [0.001, 0.01, 0.1, 1.0, 10.0, 100.0, 1000.0]}  svr_best3 = GridSearchCV(svr_best2.best_estimator_, param_grid=kn_param_grid3, cv=5,  scoring=**"neg_root_mean_squared_error"**,  n_jobs=-1,  verbose=0)  svr_best3.fit(feature_train, target_train)   **return** svr_best3, svr_best3.best_estimator_ |
| --- |

**3. Materials preparation**

All cathode materials were synthesized via sol-gel method. Firstly, critic acid (Aladdin, AR, ≥99.5%) was dissolved in 40 ml deionized water to perform as carbon source and chelating agent. Then, NH_4_VO_3_ (Aldrich, AR 99%) was added to the solution above and stirred under 60°C until turning to green. Afterwards, stoichiometric transition metal source were added to the solution, including CH_3_COONa (Aldrich, AR 99%), MnC_4_H_6_O_4_·4H_2_O (Aldrich, AR 99%), Al(NO_3_)_3_·9H_2_O (Aldrich, AR 99%), Na_2_SnO_3_·3H_2_O (Aldrich, AR 99%), C_10_H_14_MoO_6_ (Aldrich, AR 99%), H_2_N_3_O_10_Sc (Aldrich, AR 99%), CoH_12_N_2_O_12_ (Aldrich, AR 99%), C_9_H_19_NO_3_Ti (Aldrich, AR 80%), Zr(NO_3_)_4_·5H_2_O (Aldrich, AR 99%), H_12_N_3_O_15_Y (Aldrich, AR 99%) and Fe(NO_3_)_3_·9H_2_O (Aldrich, AR 99%). The entire solution was continuously stirred under 80°C for 12 h to obtain a homogeneous mixture and then dried in oven at 120°C overnight to get precursor powders. After grinding, precursor powders were transferred into a argon tube furnace and calcinated at 650 °C for 12 h with heating rate of 3 °C min^-1^.

**4. Characterization and electrochemical measurement**

To identify the crystal structure of the samples, X-ray diffraction (XRD)analysis was performed using an X-ray diffractometer (Bruker D8). Thermal gravimetric analysis (TGA) of the sample was carried out in a Mettler Thermogravimetric Analyzer at a heating rate of 10 ℃ min^-1^ in air. The inductively coupled plasma-optical emission spectroscopy (ICP, 700 DV) was carried out to confirm elements composition of samples. The morphology and microstructure of sample was researched using scanning electron microscopy (SEM) (Hitachi S4800, 10kV) and transmission electron microscopy (TEM)(ISM-2100F). The mapping of energy dispersive spectral elements was carried out in TEM mode. X-ray photoelectron spectroscopy (XPS) measurements were carried out on a Therma ESCALAB 250 instrument equipped with a monochromatic Al-Ka source.

Electrochemical experiments were conducted in CR2032 coin-type cells. The active materials, acetylene black and polyvinylidene fluoride (PVDF) were coated onto Al foil with the mass ratio of 7:2:1 using N-methyl-2-pyrrolidone (NMP) as the solvent and dried under vacuum at 80°C for 24 h to make a working electrode. The mass loading of active materials is about 2~3 mg cm^-2^. Sodium metal foil was used as the counter and reference electrode, and glass fiber as the separator. The electrolyte consisted of a solution of 1M NaClO_4_ in propylene carbonate (PC), ethylene carbonate (EC) and fluoroethylene carbonate (FEC) (10:10:1 by volume). All cells were assembled in an argon-filled glovebox ([H_2_O] and [O_2_] < 0.1 ppm).

Electrochemical measurements of cells were conducted on an electrochemical workstation (ChenHua Instruments, Inc., Model CHI 660E). The cells were Galvano statically charged and discharged on a LAND CT-2001A (Wuhan, China) and Neware CT4008Tn (Shenzhen, China) battery test system.

**5. Calculation method**

**5.1 DFT calculation**

All the density functional theory (DFT) calculations were carried out using the Vienna Ab initio Simulation Package (VASP). The exchange–correlation effects were handled using the generalized gradient approximation (GGA) of the Perdew-Burke-Ernzerhof (PBE). The interactions between core and valence electrons were described by the projector augmented wave (PAW) method. The structure relaxations were performed with a 480 eV plane-wave cutoff energy. The convergence criteria of supercell optimization for energy and force were set as 10^-5^ eV and 0.02 eV Å^−1^, respectively. And The energy barriers for Na ion diffusion in the structure were computed by the nudged elastic band (CI-NEB) method with 3 images.

**5.2 Bond valence site energy (BVSE) calculations**

To understand the migration path of Na ions in this structure, BVSE calculations were performed with the soft BV program using structural models obtained from the Rietveld refinement. The energies of different Na sites in the crystal structure were calculated against a 3D grid of points with 0.15 Å resolution using the transferable Morse-type soft BV force field. Na-ion migration pathways were identified with the regions of low BVSE.


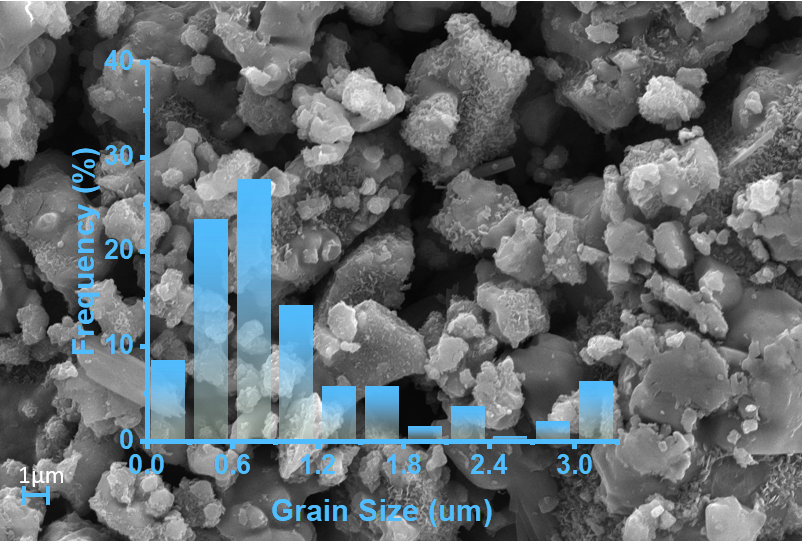


**Figure S1** SEM images (the inset is the particle size distribution curve of as-prepared NFMVTAP).


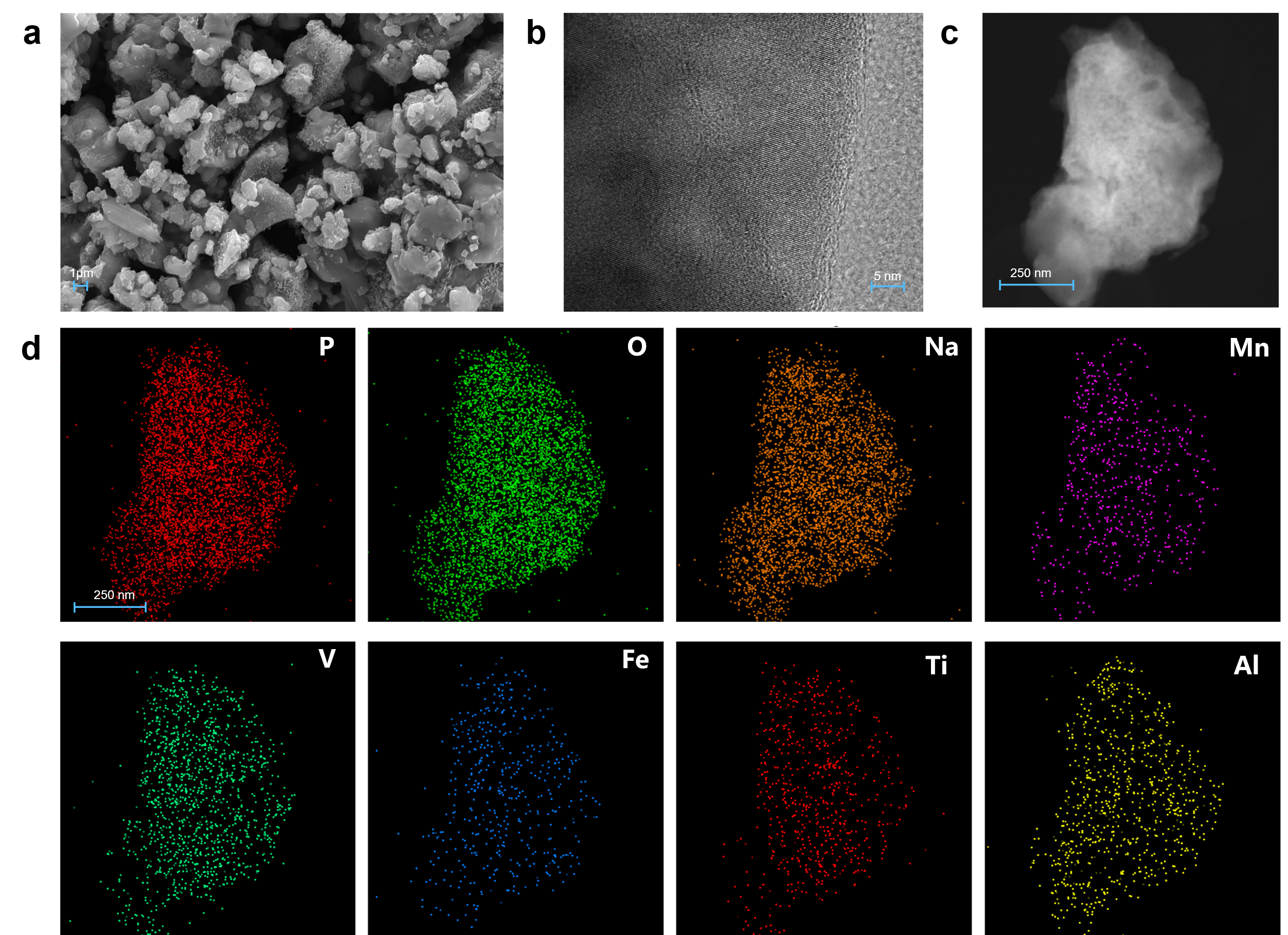


**Figure S2** a) SEM image (the inset is the particle size distribution curve of as-prepared NFMVTAP). b) High-resolution TEM image. c, d) HAADF STEM image and corresponding EDS mapping results for NFMVTAP.


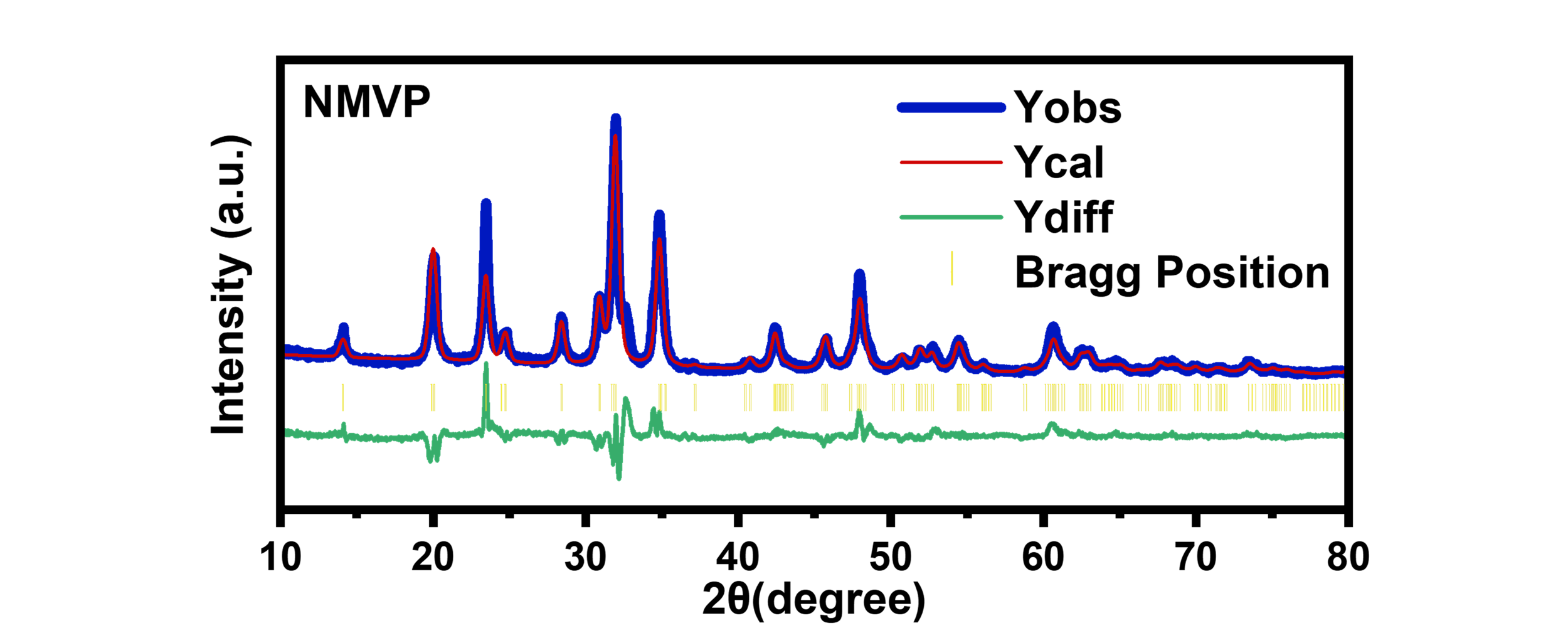


**Figure S3** XRD pattern and Rietveld refinement of NMVP.


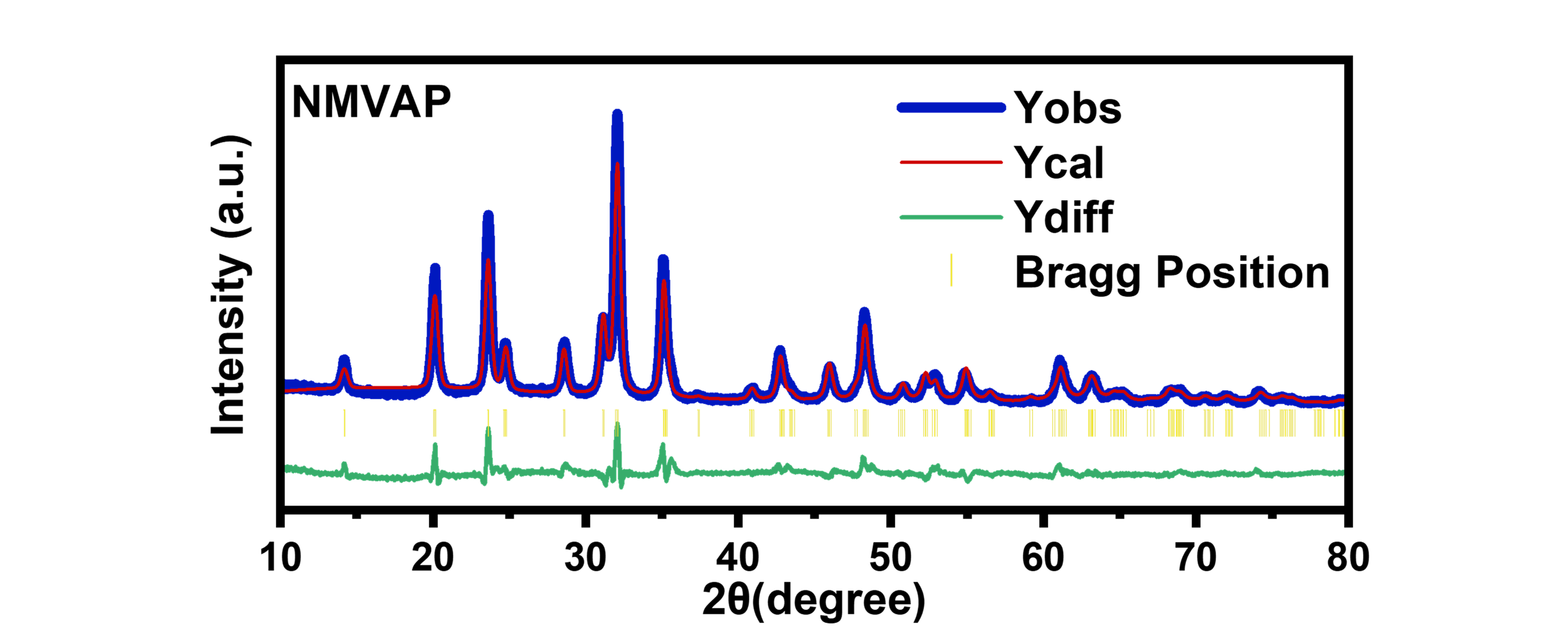


**Figure S4** XRD pattern and Rietveld refinement of NMVAP.


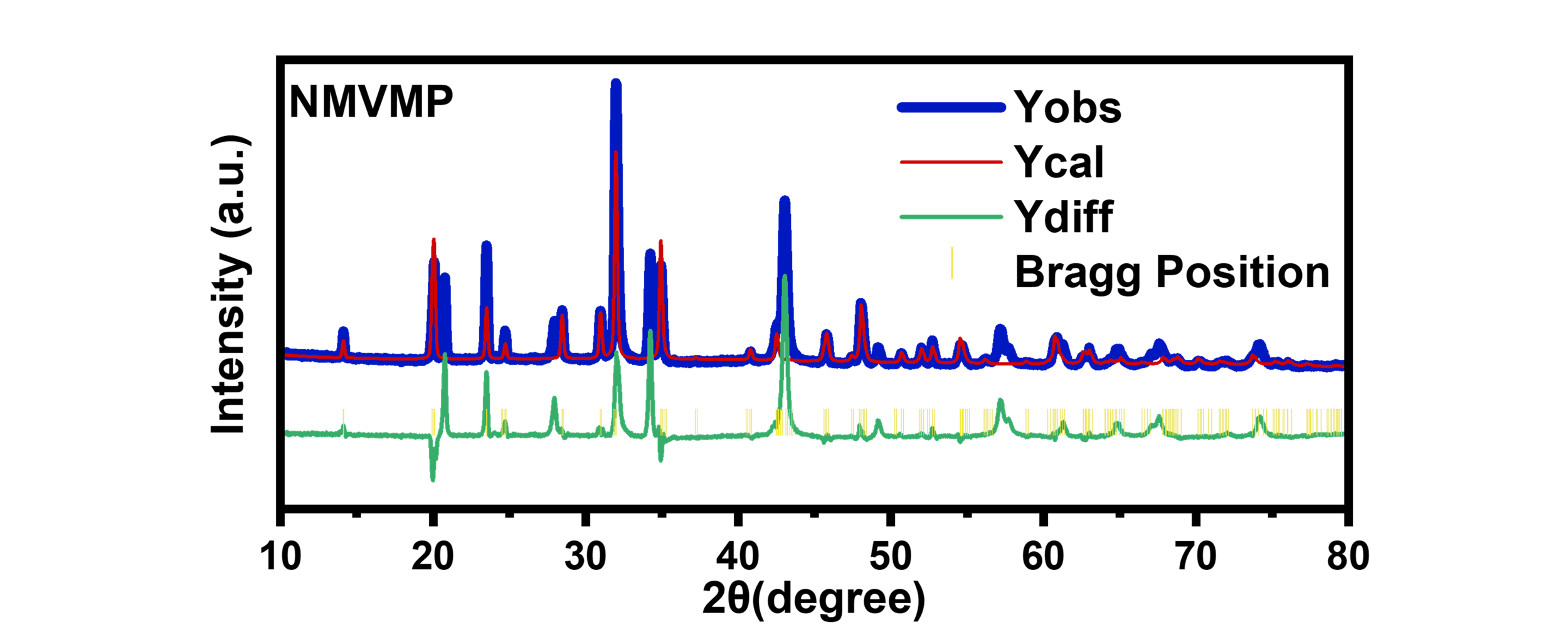


**Figure S5** XRD pattern and Rietveld refinement of NMVMP.


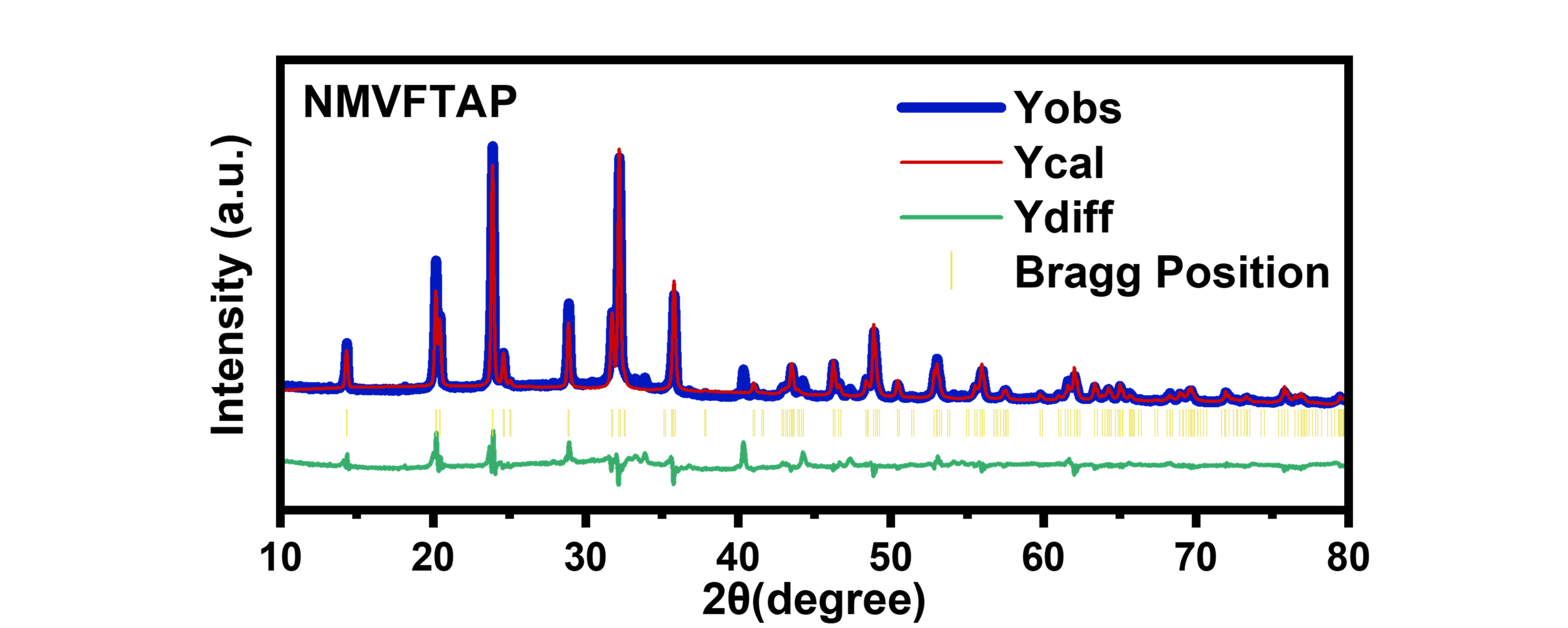


**Figure S6** XRD pattern and Rietveld refinement of NMVFTAP.


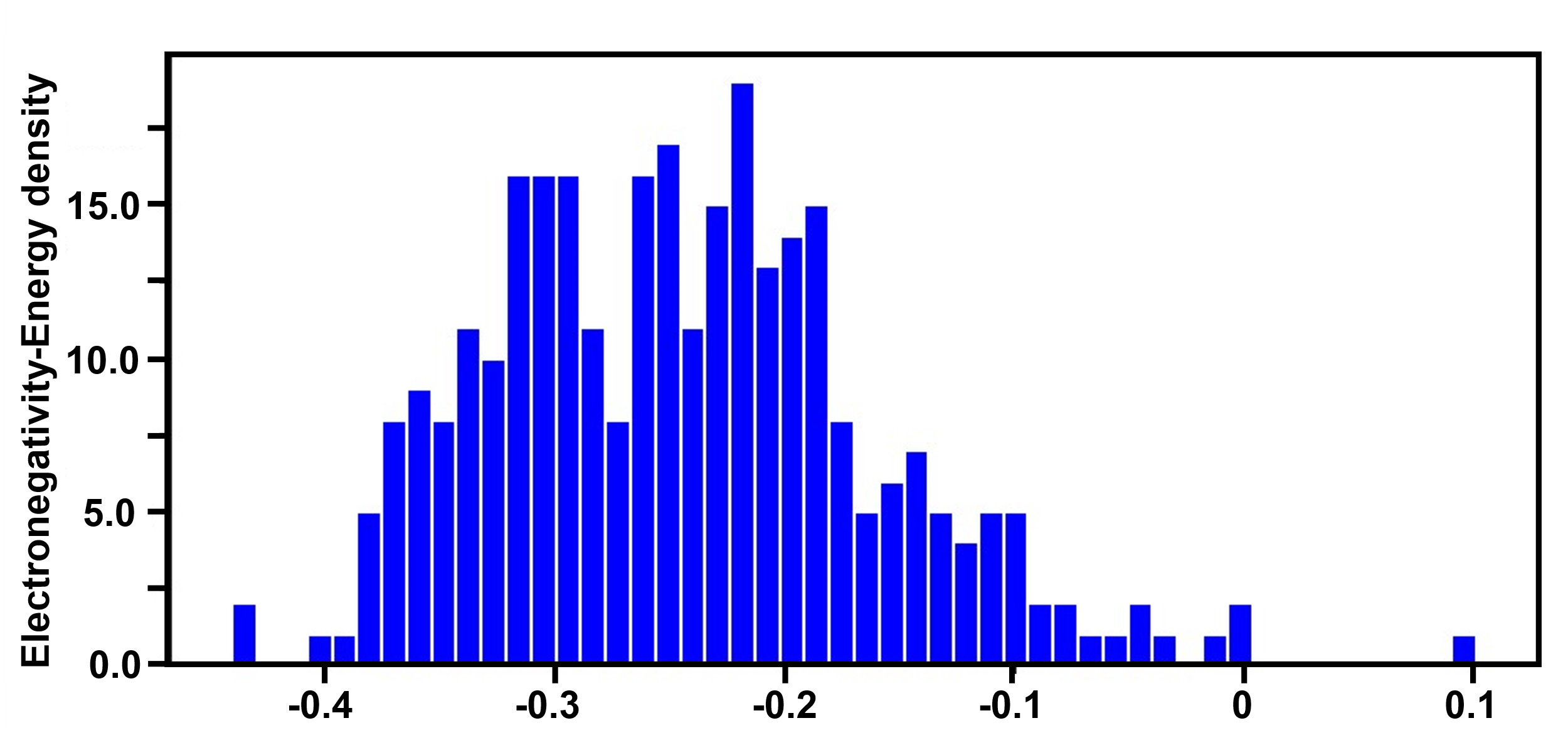


**Figure S7** Correlation distribution between Electronegativity and Energy Density for 50 randomly selected samples over 300 iterations.


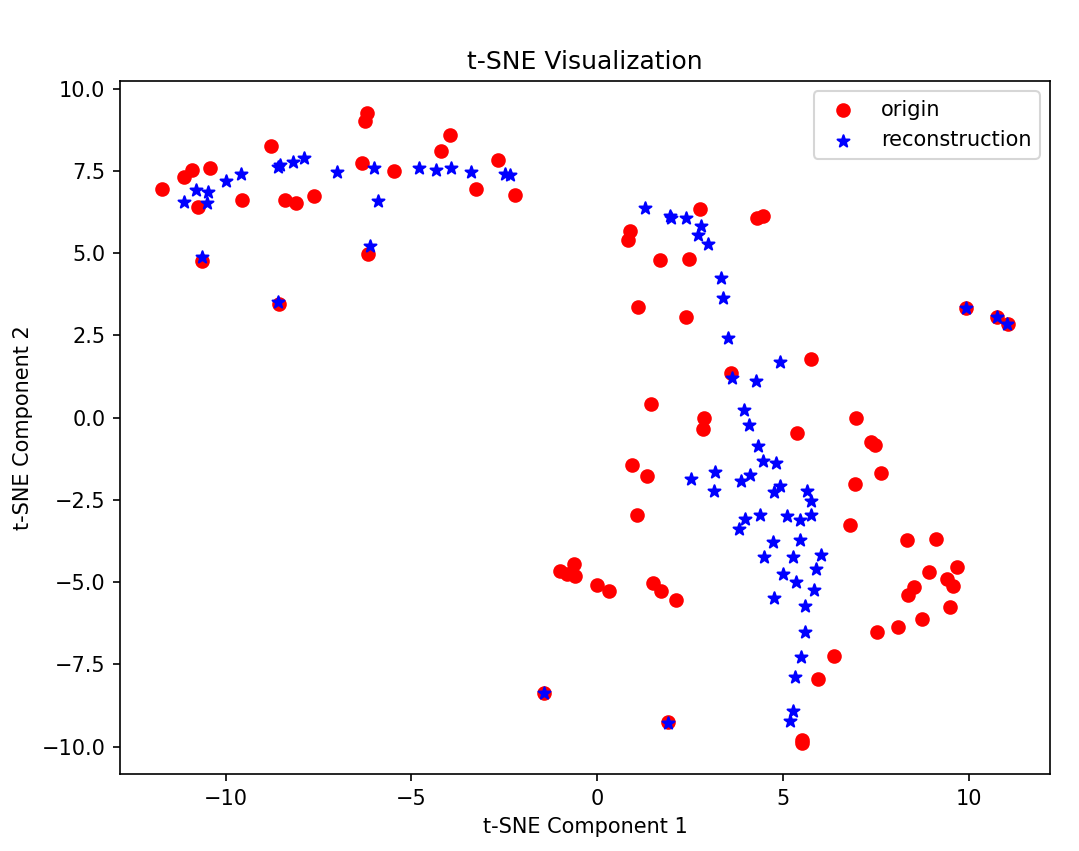


**Fig S8** Visualization of the t-SNE reconstructed after compressing the number of variables from 9 to 4 based on the autoencoder.


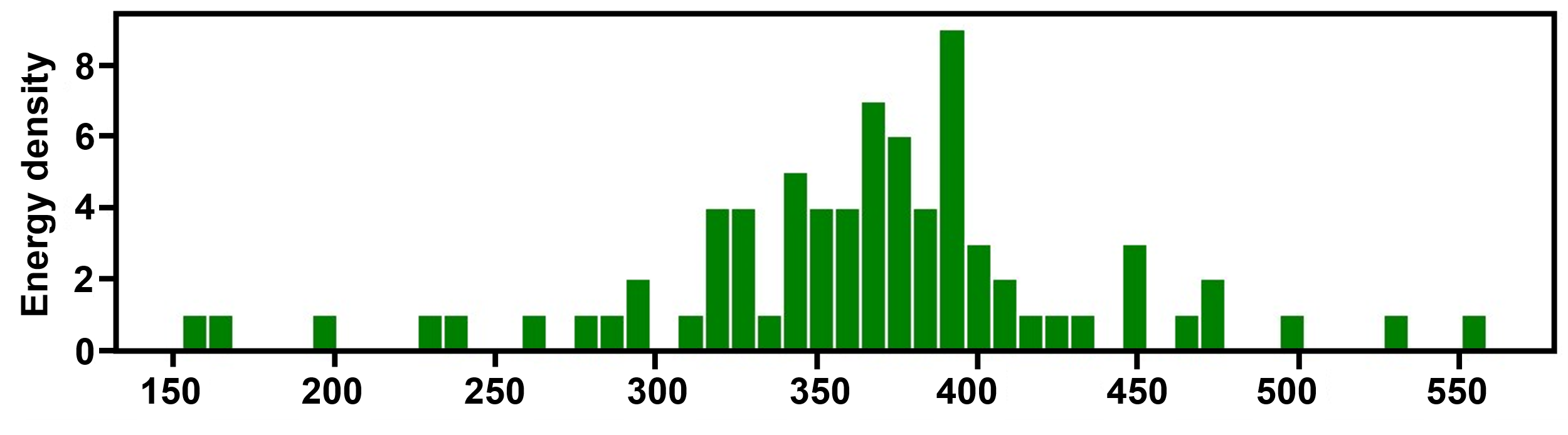


**Figure S9** Energy density distribution.

**Figure S10** The structure of AttenBNN model.


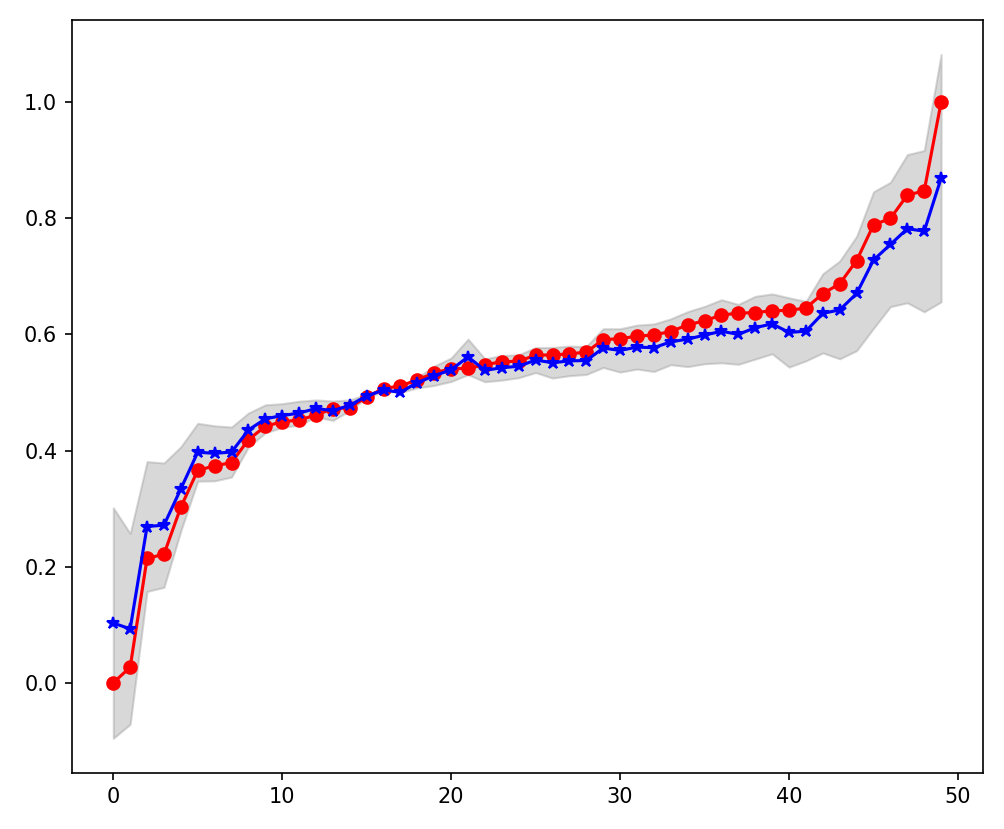

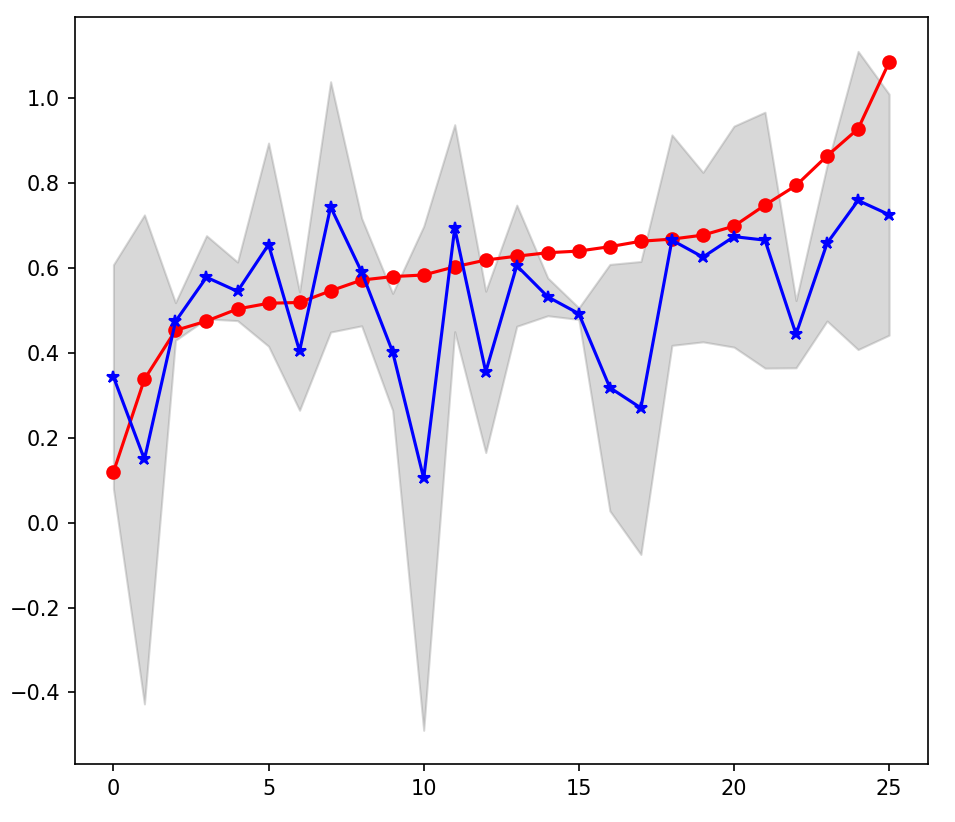


（a）AttenBNN training (b) AttenBNN testing

**Figure S11** Prediction intervals sorted by energy density.


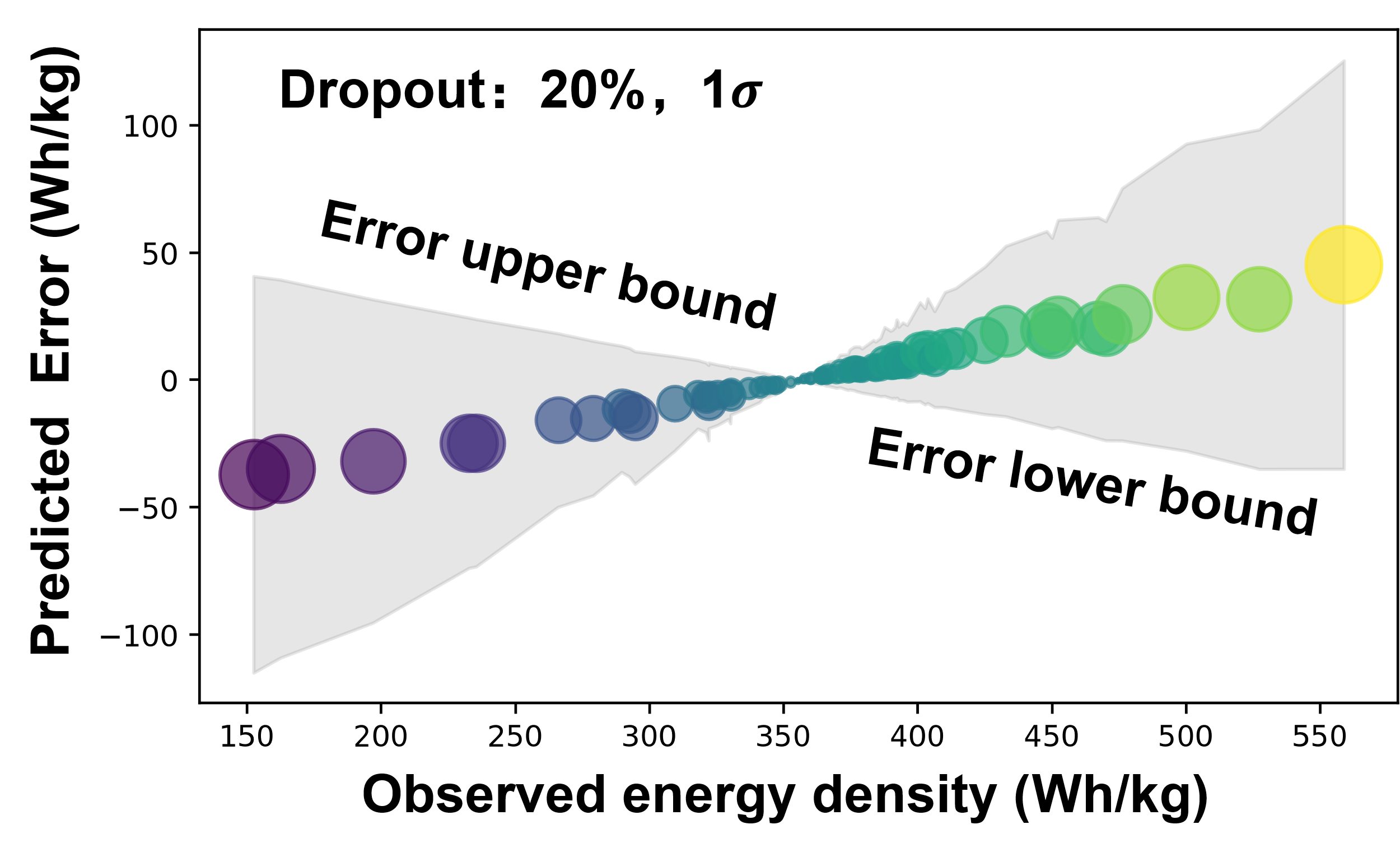


**Figure S12** Uncertainty prediction results.

**Figure S13** The optimal parameter space for each feature.


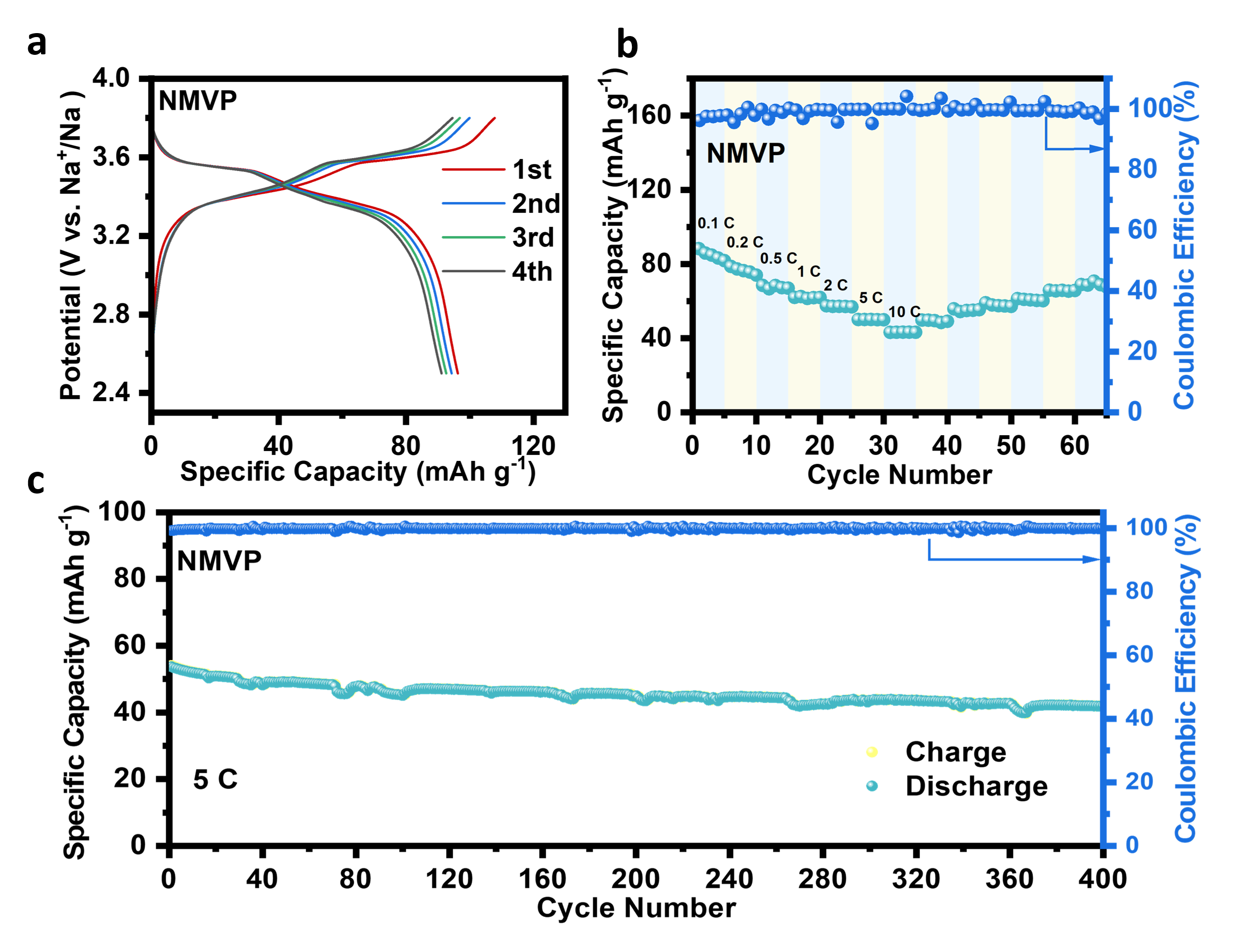


**Figure S14** Experimental results. a) Charge-discharge curves for the first four cycles of Na_4_MnV(PO_4_)_3_ b) Rate performance from 0.1 C to 10 C of Na_4_MnV(PO_4_)_3_ c) Cycling performance at 5 C for 400 cycles of Na_4_MnV(PO_4_)_3_.


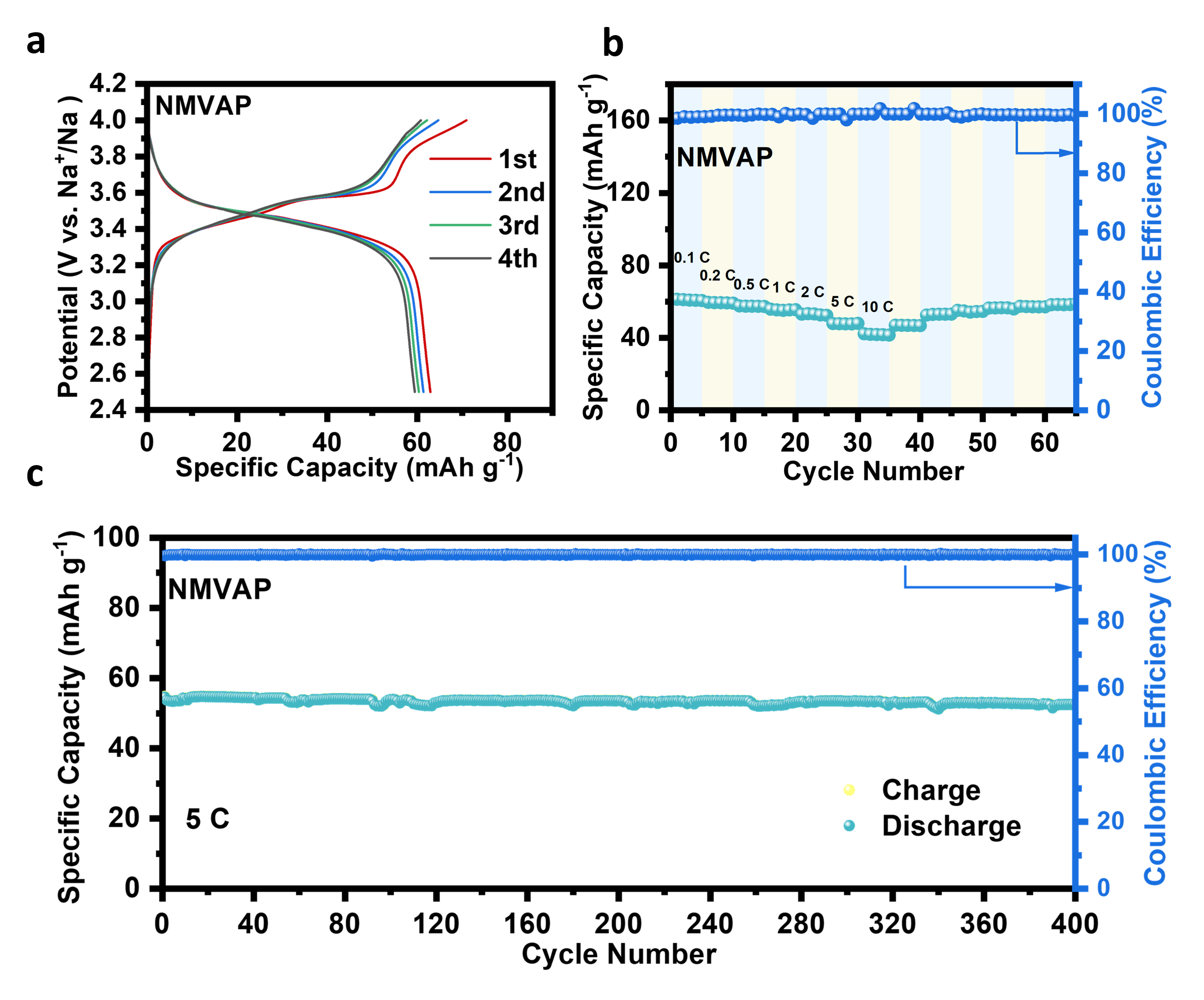


**Figure S15** Experimental results. a) Charge-discharge curves for the first four cycles of Na_11/3_Mn_2/3_V_2/3_Al_2/3_(PO_4_)_3_ b) Rate performance from 0.1 C to 10 C of Na_11/3_Mn_2/3_V_2/3_Al_2/3_(PO_4_)_3_ c) Cycling performance at 5 C for 400 cycles of Na_11/3_Mn_2/3_V_2/3_Al_2/3_(PO_4_)_3_.


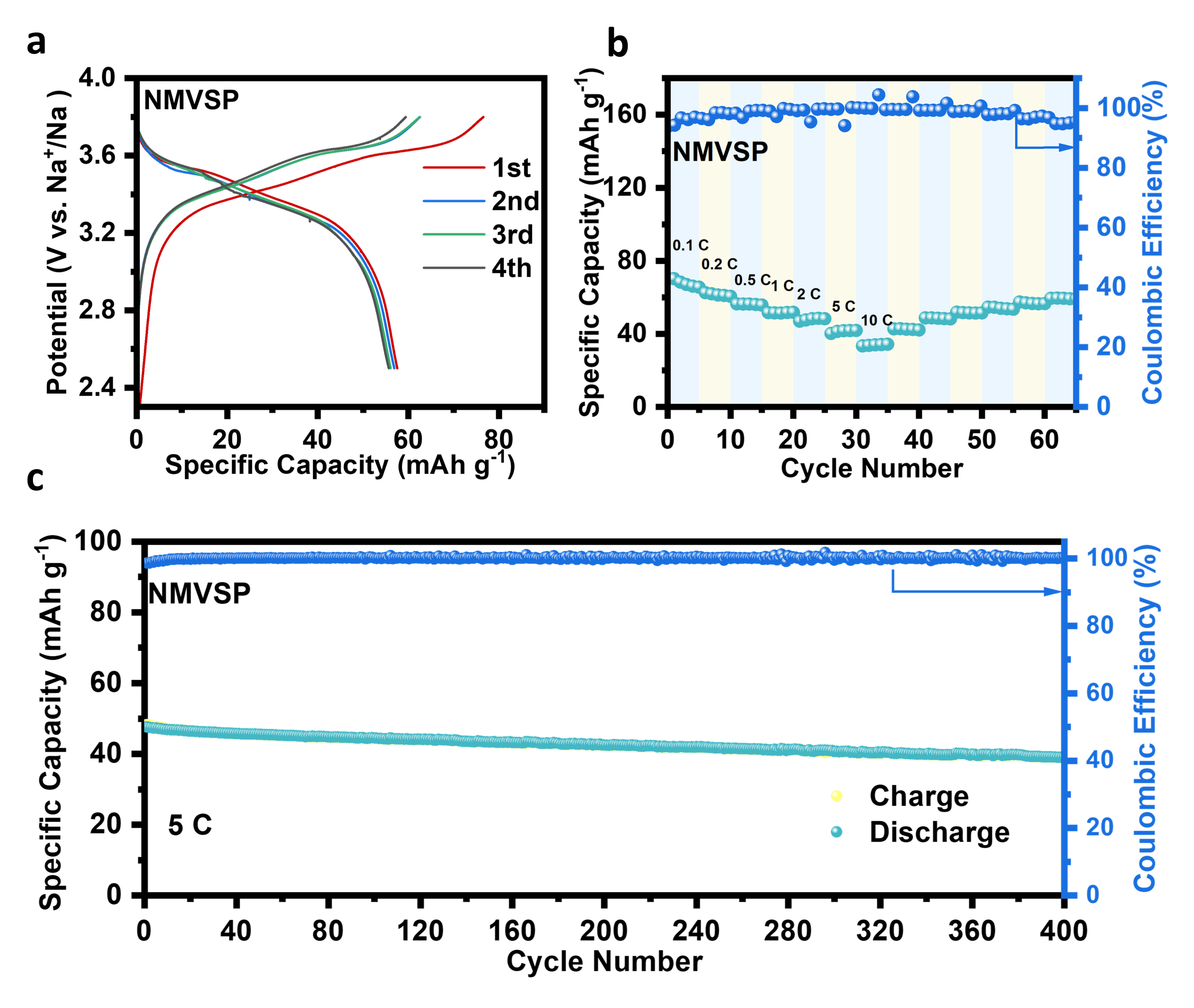


**Figure S16** Experimental results. a) Charge-discharge curves for the first four cycles of Na_11/3_Mn_2/3_V_2/3_Sc_2/3_(PO_4_)_3_ b) Rate performance from 0.1 C to 10 C of Na_11/3_Mn_2/3_V_2/3_Sc_2/3_(PO_4_)_3_ c) Cycling performance at 5 C for 400 cycles of Na_11/3_Mn_2/3_V_2/3_Sc_2/3_(PO_4_)_3_.


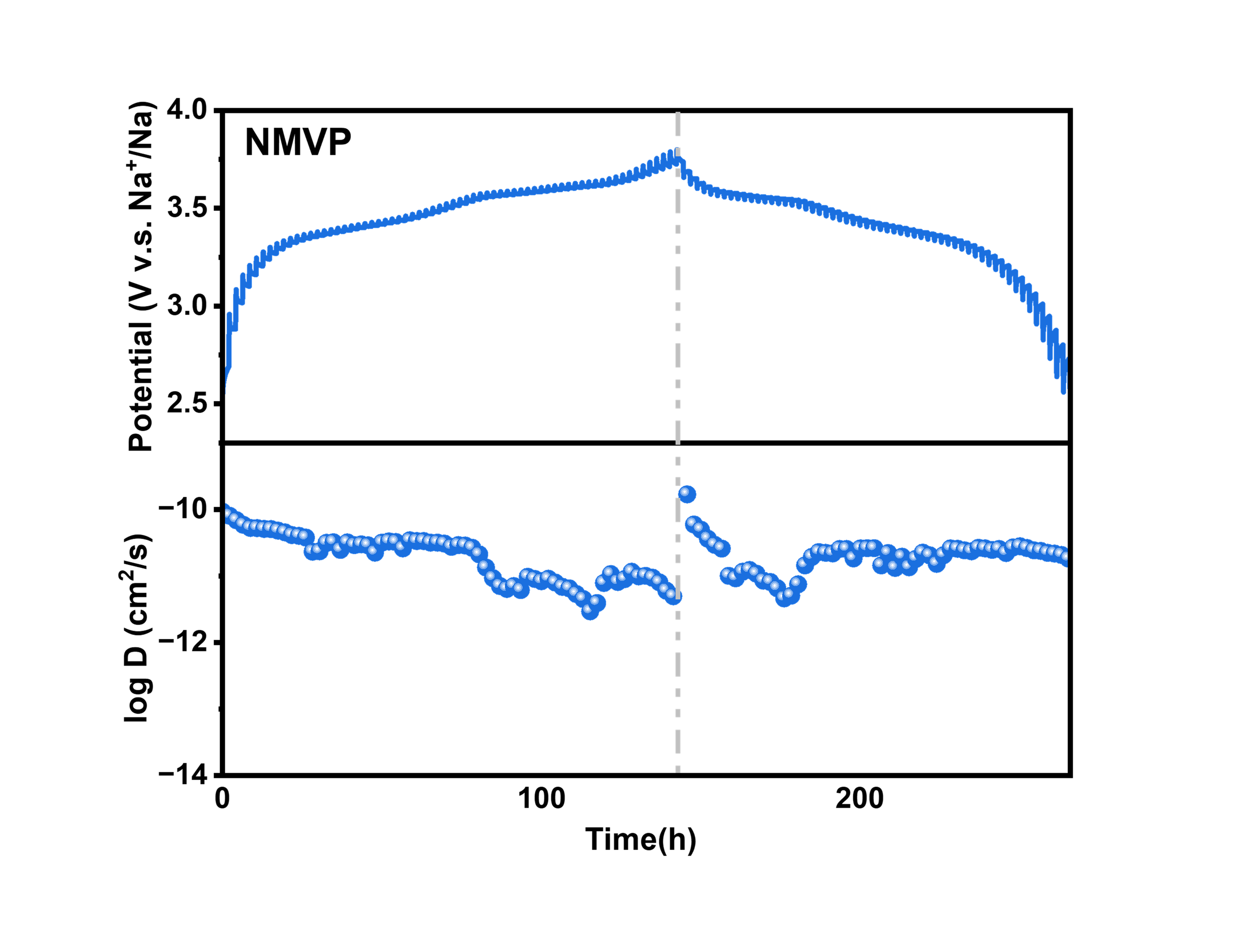


**Figure S17** The apparent Na+ diffusion coefficient of the NMVP cathode cycled at room temperature and 0.1 C.


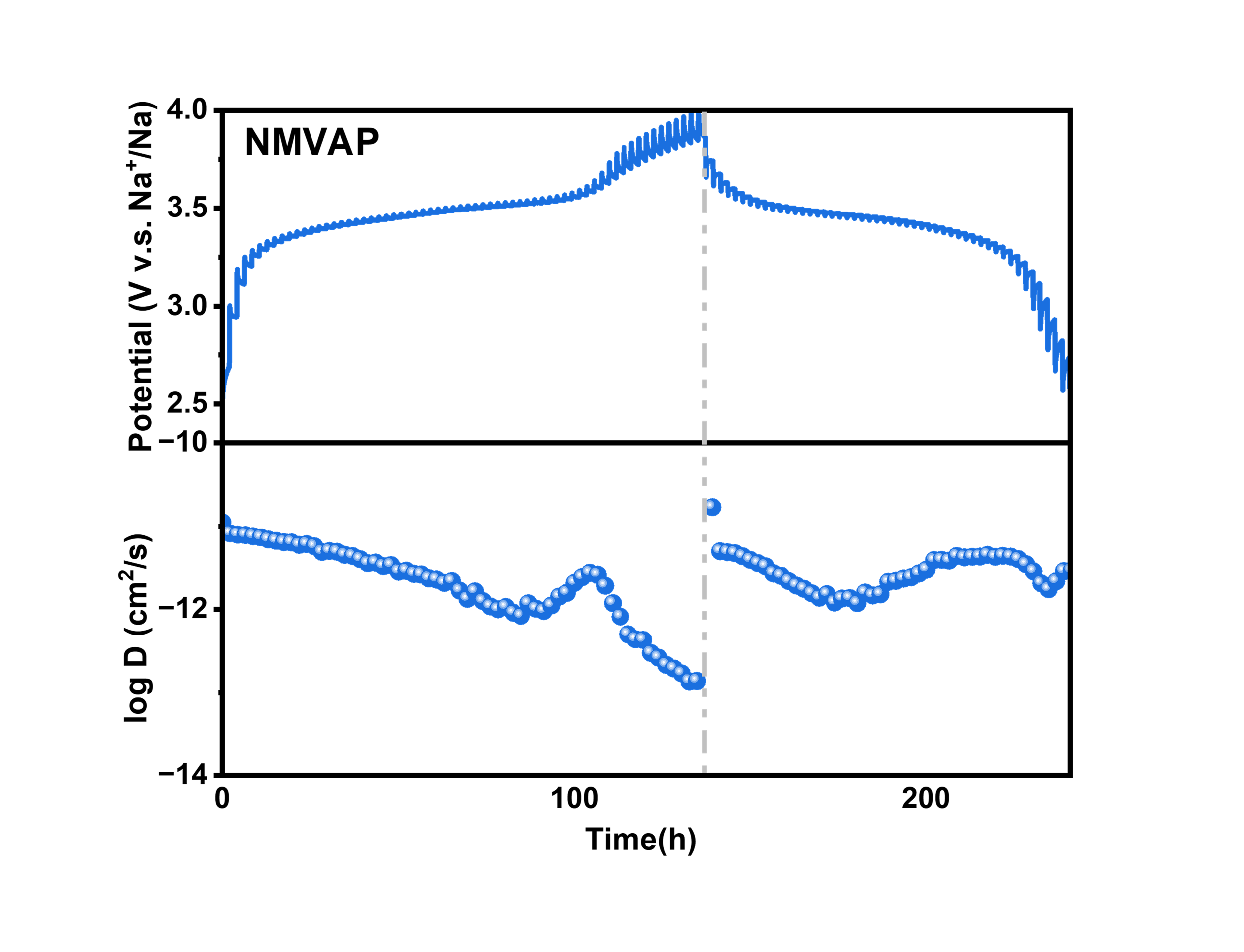


**Figure S18** The apparent Na+ diffusion coefficient of the NMVAP cathode cycled at room temperature and 0.1 C.


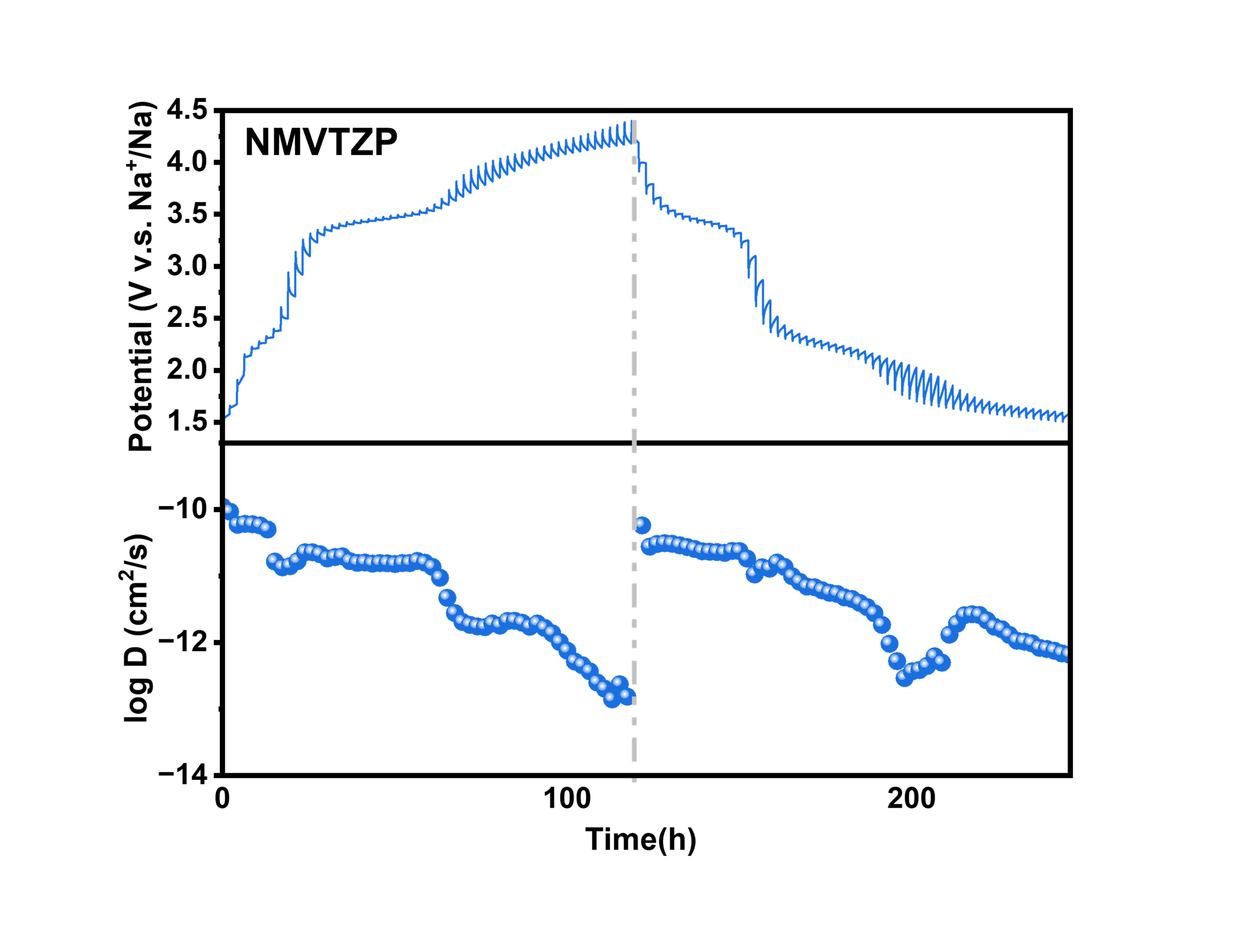


**Figure S19** The apparent Na+ diffusion coefficient of the NMVTZP cathode cycled at room temperature and 0.1 C.


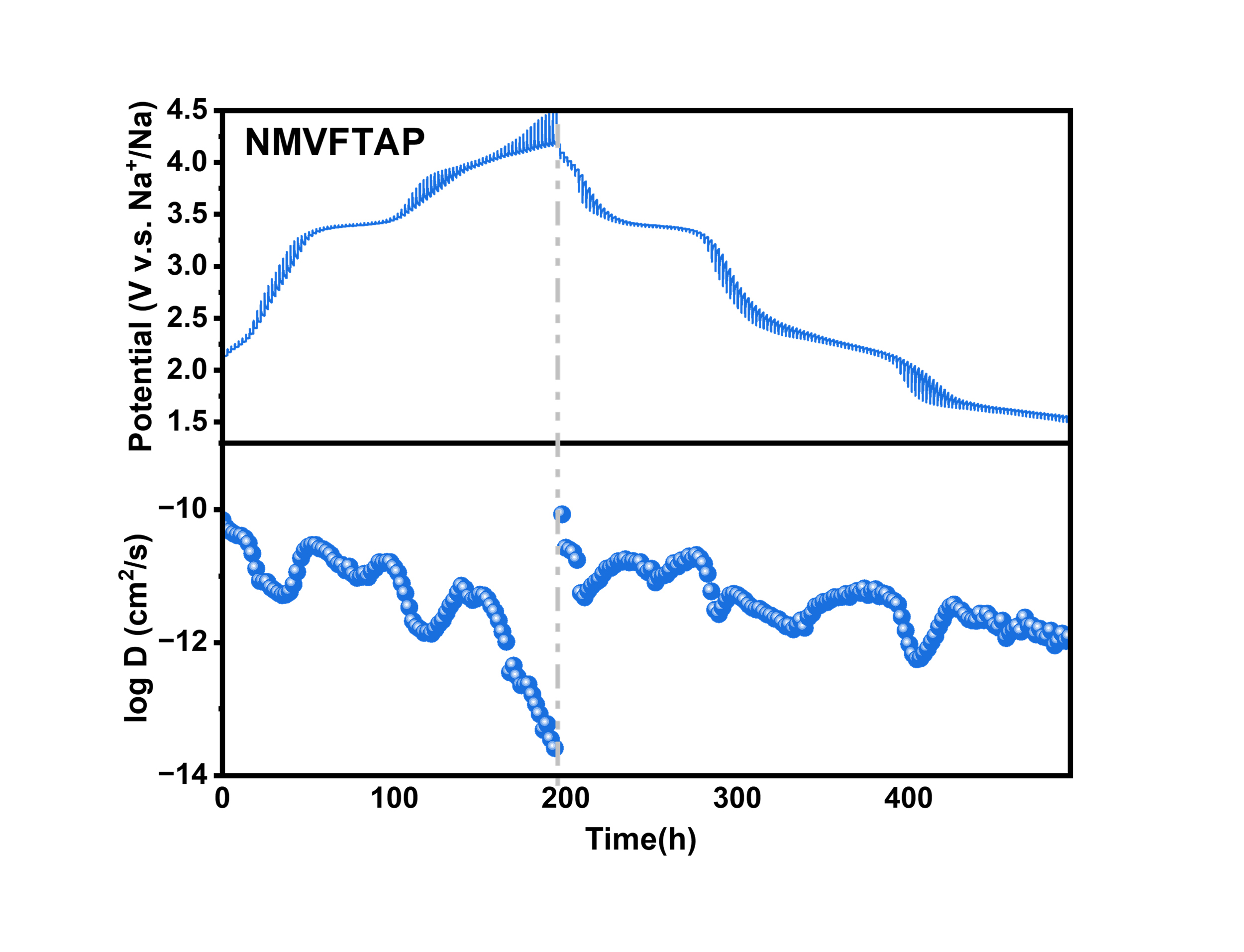


**Figure S20** The apparent Na+ diffusion coefficient of the NMVFTAP cathode cycled at room temperature and 0.1 C.


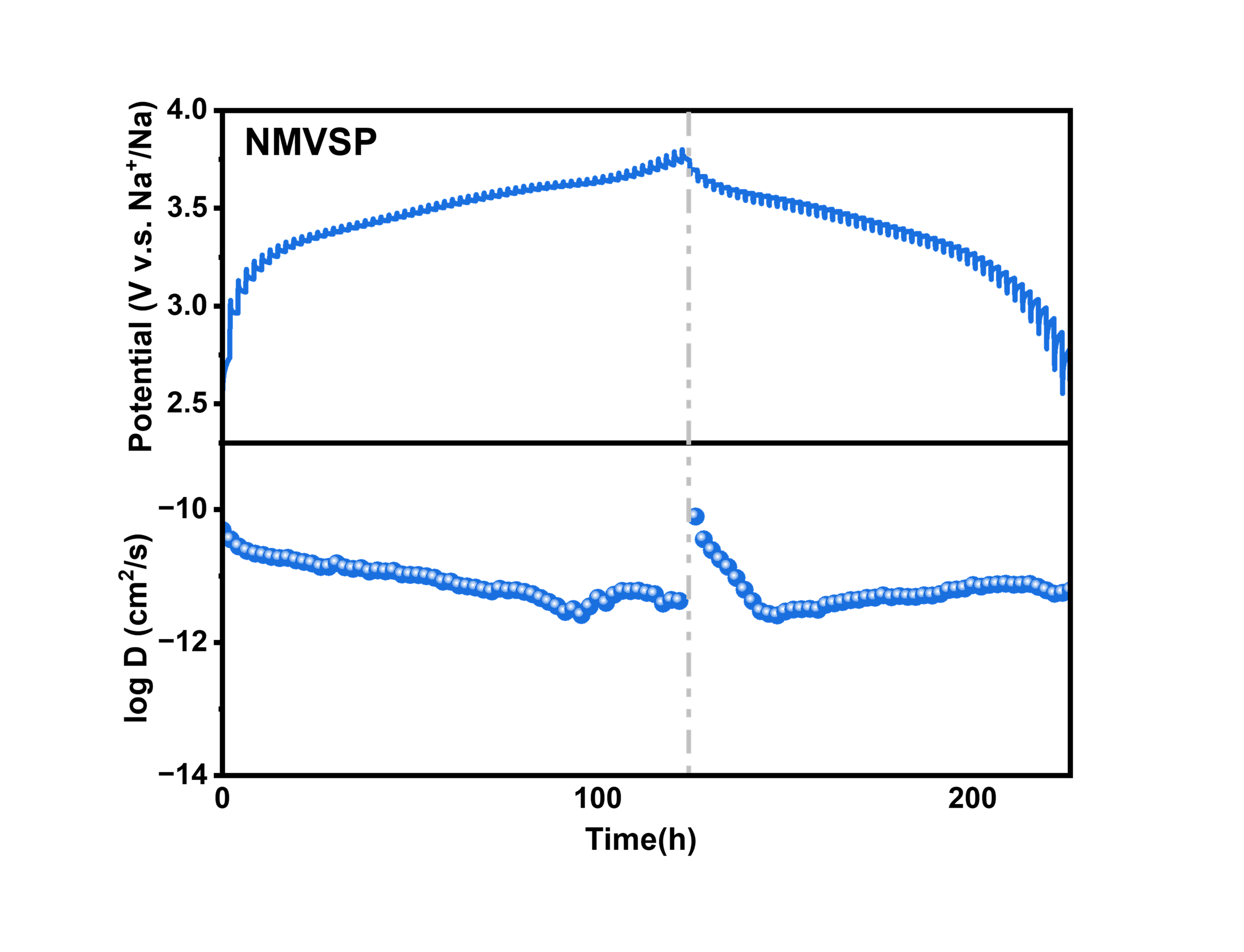


**Figure S21** The apparent Na+ diffusion coefficient of the NMVSP cathode cycled at room temperature and 0.1 C.


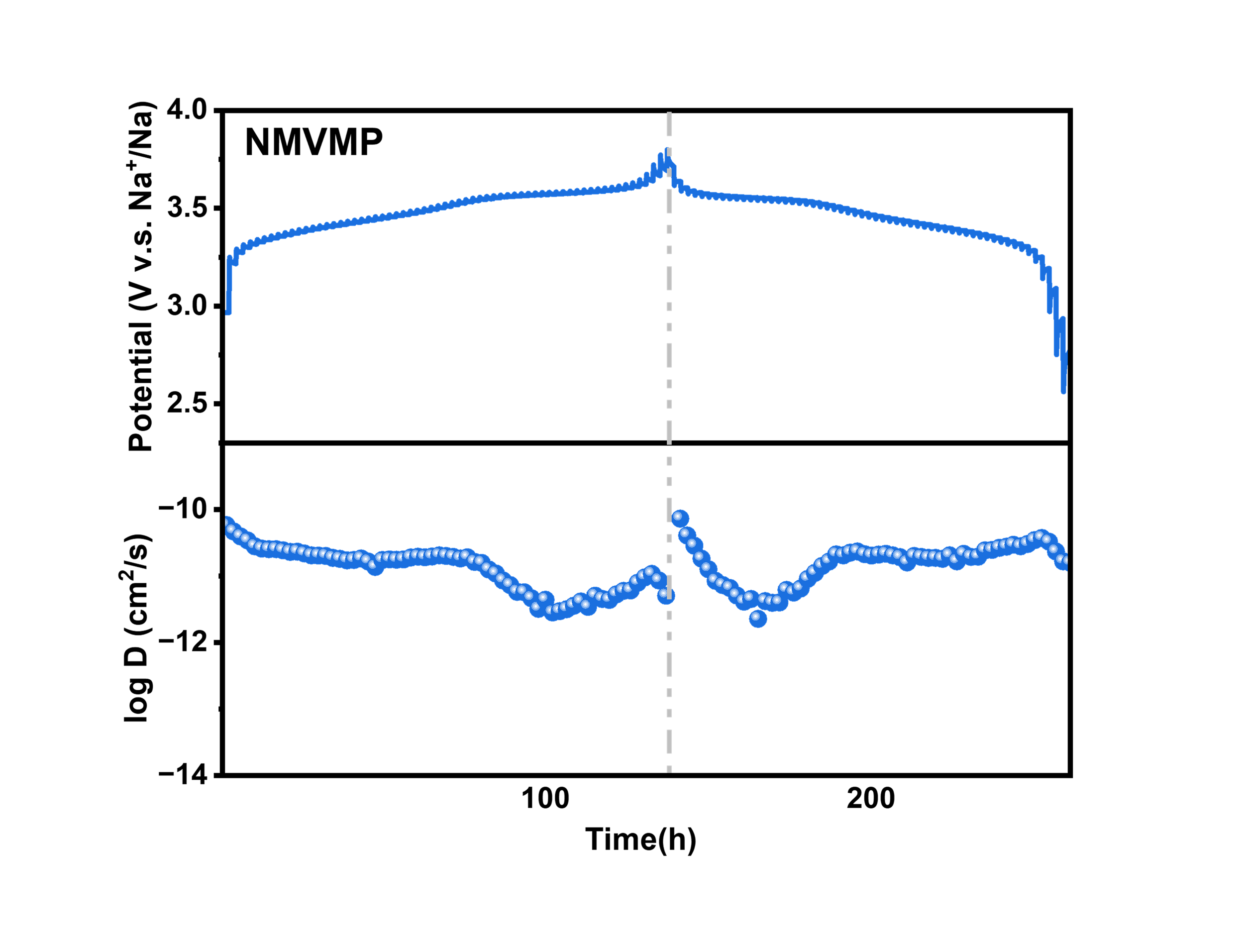


**Figure S22** The apparent Na+ diffusion coefficient of the NMVMP cathode cycled at room temperature and 0.1 C.

**
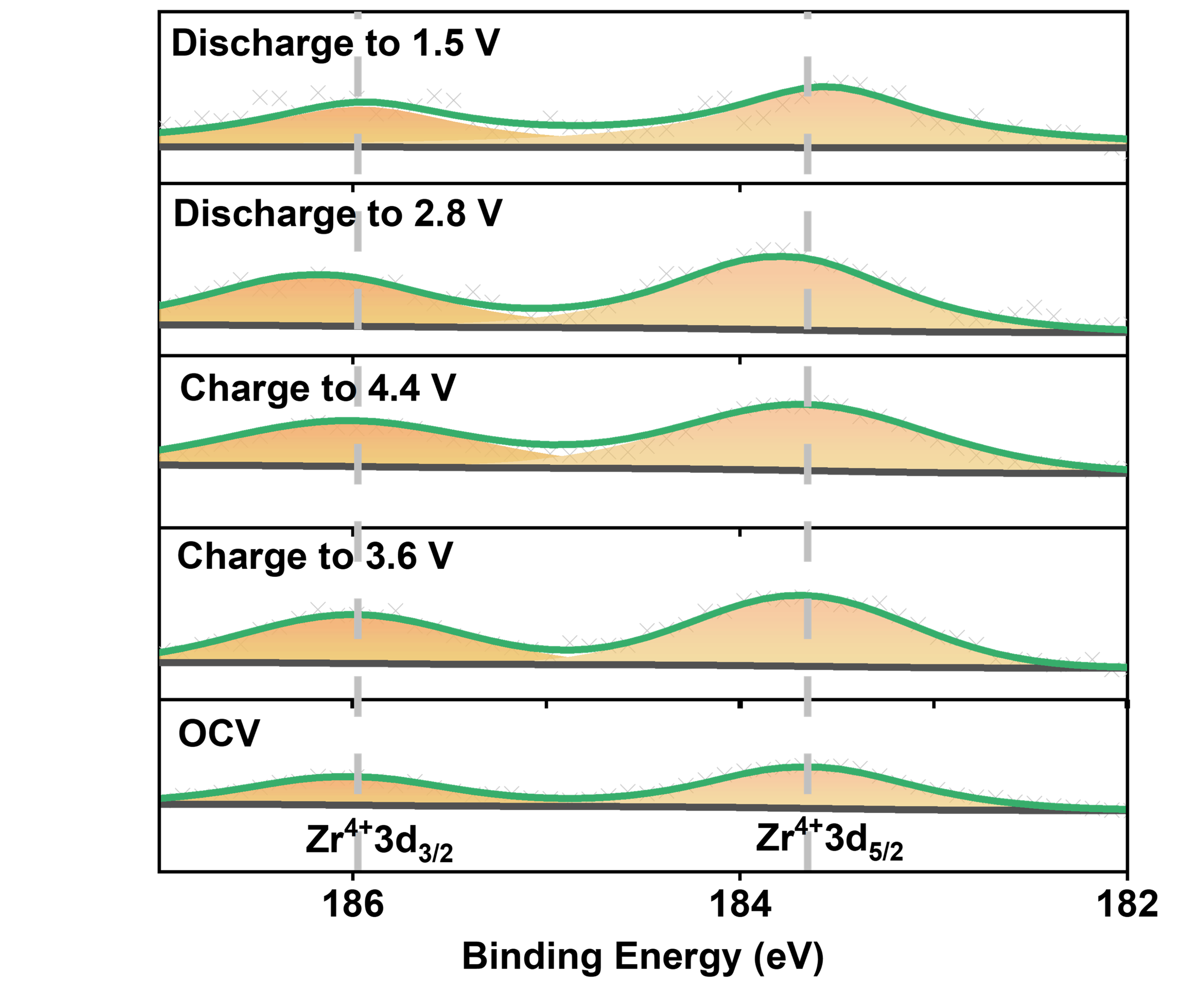
**

**Figure S23** XPS spectra of the Zr that collected from the OCV, charged to 3.6 V, charged to 4.4 V, discharged to 2.8 V, discharged to 1.5 V of the NMVTZP.

**Table S1**. Atomic ratio obtained from EDS analysis.

| Map Sum Spectrum | Atomic % |
| --- | --- |
| O | 47.48 |
| Na | 17.22 |
| P | 22.48 |
| Ti | 2.68 |
| V | 3.56 |
| Mn | 2.65 |
| Zr | 3.93 |
| Total | 100.00 |

**Table S2**. Detailed structure information of Na_3_Mn_0.5_V_0.5_Ti_0.5_Zr_0.5_(PO_4_)_3_: from Rietveld refinement from the X-ray power diffraction pattern.

| Na_3_Mn_0.5_V_0.5_Ti_0.5_Zr_0.5_(PO_4_)_3_ sample. Rhombohedral symmetry, space group *R3c*  *a*=*b*=8.807731Å, *c*=22.168936 Å, V=1,489.33 Å^3^  weighted profile R-factor, R_wp_=8.62%, R_p_=10.8%, X^2^=32% | | | | |
| --- | --- | --- | --- | --- |
| Label | X | Y | Z | Occupancy |
| Na1 | 0.3333 | 0.6667 | 0.1667 | 1.05246 |
| Na2 | 0.6667 | 0.96726 | 0.0833 | 0.66666 |
| V | 0.3333 | 0.6667 | 0.01932 | 0.25 |
| Mn | 0.3333 | 0.6667 | 0.01932 | 0.25 |
| Ti | 0.3333 | 0.6667 | 0.01932 | 0.25 |
| Zr | 0.3333 | 0.6667 | 0.01932 | 0.25 |
| P | -0.04273 | 0.3333 | 0.0833 | 1.05006 |
| O1 | 0.14193 | 0.49765 | 0.07762 | 1.01695 |
| O2 | 0.54047 | 0.8448 | -0.02643 | 0.95336 |

**Table S3**. Element property parameters.

| Transition metal ions | Electronegativity | Ionic radius /pm |
| --- | --- | --- |
| V^3+^ | 1.63 | 74 |
| Mn^2+^ | 1.55 | 80 |
| Al^3+^ | 1.61 | 50 |
| Mo^3+^ | 2.16 | 69 |
| Sc^3+^ | 1.36 | 81 |
| Fe^3+^ | 1.83 | 64 |
| Ti^3+^ | 1.54 | 76 |
| Zr^4+^ | 1.33 | 80 |

**Table S4**. Materials’ property parameters.

| Material | Electronegativity | Average  Ionic radius | Electronic affinity | Entropy |
| --- | --- | --- | --- | --- |
| Na_4_MnV(PO_4_)_3_  (NMVP) | 3.18 | 77.00 | 50.60 | 0.6939 |
| Na_11/3_Mn_2/3_V_2/3_Al_2/3_(PO_4_)_3_  (NMVAP) | 3.19 | 68.00 | 62.07 | 1.099 |
| Na_11/3_Mn_2/3_V_2/3_Mo_2/3_(PO_4_)_3_  (NMVMP) | 3.56 | 99.00 | 81.67 | 1.099 |
| Na_11/3_Mn_2/3_V_2/3_Sc_2/3_(PO_4_)_3_  (NMVSP) | 3.03 | 78.33 | 45.80 | 1.099 |
| Na_3_Mn_0.5_V_0.5_Ti_0.5_Zr_0.5_(PO_4_)_3_  (NMVTZP) | 3.025 | 77.5 | 49.65 | 1.386 |
| Na_3.4_Fe_0.4_Mn_0.4_V_0.4_Ti_0.4_Al_0.4_(PO_4_)_3_  (NMVFTAP) | 3.264 | 68.80 | 46.56 | 1.609 |

**Table S5**. Summary of electrochemical performance for high energy density NASICON cathode electrodes.

| Cathodes | Capacity  mAh g^-1^ | Cycling Performance | Ave. Voltage | Energy Density (Wh kg^-1^) |
| --- | --- | --- | --- | --- |
| Na_4_VMn(PO_4_)_3_[62] | 112.0 (20 mA g^-1^) | 85.1%, 1200, 1 A g^-1^ | 3.5 | 392 |
| Na_4_MnTi(PO_4_)_3_[63] | 114.0 (0.2 C) | 76.6%, 800, 1 C | 3.59 | 410 |
| Na_2_VTi(PO_4_)_3_[64] | 147.0 (0.1 C) | 77.0%, 500, 10 C | 2.42 | 356 |
| Na_4_MnCr(PO_4_)_3_[65] | 109.7 (0.01 A g^-1^) | 54.2%, 500, 0.5 A g^-1^ | 3.8 | 422 |
| Na_4_Cr_0.7_Fe_0.4_Mn_0.3_  V_0.3_Al_0.2_(PO_4_)_3_ [66] | 165.0 (0.1 C) | 92.4%, 100, 5 C | 2.84 | ~470 |
| Na_3.359_V_1.421_Zn_0.351_  (GaCrAlIn)_0.06_(PO_4_)_3_[67] | 193.4 (0.1 C) | 94.8%, 200, 1 C | 2.81 | 544 |
| Na_3_(VMnTi)_2/3_(PO_4_)_3_[68] | 148.0 (50 mA g^-1^) | 88.3%, 1000, 0.5 A g^-1^ | 2.65 | 393 |
| **This work** | 148.3 (0.1 C) | 78.1%, 400, 5 C  93.4%, 100, 5 C | 3.14 | 465 |
